# Japanese encephalitis virus persists in the human reproductive epithelium and porcine reproductive tissues

**DOI:** 10.1371/journal.pntd.0010656

**Published:** 2022-07-29

**Authors:** Subash Chapagain, Prince Pal Singh, Khanh Le, David Safronetz, Heidi Wood, Uladzimir Karniychuk

**Affiliations:** 1 Vaccine and Infectious Disease Organization (VIDO), University of Saskatchewan, Saskatoon, Canada; 2 Department of Veterinary Microbiology, Western College of Veterinary Medicine, University of Saskatchewan, Saskatoon, Canada; 3 School of Public Health, University of Saskatchewan, Saskatoon, Canada; 4 The National Microbiology Laboratory, Public Health Agency of Canada, Winnipeg, Canada; London School of Hygiene & Tropical Medicine, UNITED KINGDOM

## Abstract

Japanese encephalitis virus (JEV) is the emerging and geographically expanding flavivirus and the major causative agent of encephalitis in humans in Asia. There are risks of JEV introduction into the Americas given a large population of amplifying hosts—pigs and wild boars, and insect vectors—*Culex* mosquitoes. There are emerging concerns about vector-free ways of flavivirus transmission, for example sexual and transplacental Zika virus transmissions, which may change flavivirus epidemiology and expand the geographical range to territories with no insect vectors. It is unknown whether JEV has tropism in the female lower reproductive tract and the potential for sexual transmission in humans. While clinical outcomes of transplacental JEV infection are described in humans and pigs, cellular targets and tissue tropism in the upper reproductive tract are also unknown. Here, we studied JEV infection phenotypes and host transcriptional responses in human reproductive epithelial cells. We found that JEV caused persistent infection and cytopathology in the vaginal epithelium, endometrial epithelium, and trophoblast. Human vaginal epithelial cells infected with JEV had altered transcriptional responses associated with inflammation and disruption of epithelial barrier function. Also, using pigs—the native amplifying host for JEV, we confirmed JEV tropism in the female lower and upper reproductive tracts. We discovered that JEV persists in the vaginal mucosa for at least 28 days and pigs shed the virus in vaginal secretions. We also found JEV persistence in the endometrium and placenta with transplacental and fetal infections. Altogether, we discovered that JEV targets the vaginal epithelium and has the potential for sexual transmission in humans. We also contributed to a better understanding of JEV pathogenesis during transplacental infection. Further studies are needed to better understand the interactions of JEV with reproductive tissues, how persistent infection affects female reproductive functions, and the risks for non-vector transmission.

## Author summary

Emerging viruses—newly discovered or with increasing disease incidence—pose a constant threat to public health. The most recent examples of devastating outbreaks of emerging viruses are Ebola virus, new coronaviruses, and Zika virus epidemics. Japanese encephalitis virus (JEV) is the emerging flavivirus related to Zika virus; it is the most important cause of brain infections in Asia that may cause death and severe neurological sequela in patients. Almost half of the world’s population lives in territories where JEV is permanently circulating. Like Zika virus, JEV is transmitted to humans via mosquito bites. However, there are emerging concerns about alternative beyond mosquito-borne ways of flavivirus transmission which may expand the geographical pathogen range to territories with no insect vectors. For example, sexual and transplacental Zika virus transmissions with replication in human vaginal epithelial cells, placenta, and fetuses have been described. Here, we questioned whether JEV also has potential for sexual transmission and studied its tropism in the human female reproductive epithelium—the primary barrier cells of the reproductive system. Also, using pigs—the native amplifying host for JEV, we studied JEV infection in the female lower and upper reproductive tract tissues. We discovered that JEV replicates in different reproductive epithelial cells and tissues of both humans and pigs for a long time, causing molecular and clinical pathology. Thus, further studies are needed to better understand the interactions of JEV with reproductive tissues, how persistent infection affects female reproductive functions, and the risks for non-mosquito transmissions.

## Introduction

Japanese encephalitis virus (JEV) is a zoonotic flavivirus transmitted by *Culex* mosquitoes. While efficient vaccines are available [1], JEV is the major causative agent of encephalitis in humans in the Asia-Pacific region [2,3], with an estimated 68,000 cases reported annually and 15,000 deaths; statistics, however, are most probably underestimated [4]. Though humans are dead-end hosts of JEV and most human infections are asymptomatic, around 20–30% of clinical infections are fatal, and 30–50% of the survivors develop prolonged or life-long neurological sequelae [2]. There is no specific cure for the disease; treatment is only supportive to mitigate disease outcomes. The geographic range of emerging JEV keeps expanding from the South of Russia to Australia, including Japan, Eastern China, India, and South-East Asia. For example, the first major outbreak of JEV in Australia with infections in pig herds and human deaths unfolded in February 2022. Also, travel-related severe cases of JEV-induced encephalitis are reported around the globe [5]. There is a concern that JEV can be introduced into North America given a large population of amplifying hosts—pigs and wild boars; susceptible *Culex* mosquitoes are also ubiquitous [6,7].

There are emerging concerns about alternative, beyond vector-borne ways of flavivirus transmission which may change flavivirus epidemiology and expand the geographical range to territories with no insect vectors. For example, it was thought that JEV transmission occurred only through mosquitoes, but a recent study demonstrated that JEV is transmitted from pig to pig, suggesting vector-free ways for the virus to spread [8]. Following studies confirmed efficient JEV replication in porcine and human nasal epithelial cells [9,10] and virus oronasal shedding in pigs [11,12] that can enable contact transmission. Another example of vector-free flavivirus transmission is sexual Zika virus transmission. Many sexual transmissions of Zika virus have been described, including male-to-male, male-to-female, female-to-male transmissions [13–15], and fetal infection with congenital Zika syndrome after sexual transmission in mothers [16]. Rodent and non-human primate models also supported replication in reproductive tissues and sexual transmission of Zika virus [17–23]. In humans, Zika virus has been detected in vaginal secretions [24–26] with replication in vaginal epithelial cells [18]. It is unknown, however, whether JEV also has tropism in the vaginal epithelium and the potential for sexual transmission in humans. To probe this likelihood, in the present study, we studied JEV infection phenotypes and host transcriptional responses in human primary vaginal epithelial cells. Also, we used pigs—the native amplifying host for JEV and studied infection in vaginal tissues and vaginal virus shedding.

Another example of vector-free flavivirus transmission is transplacental Zika virus transmission. Zoonotic flaviviruses were thought to primarily impact human non-reproductive tissues. However, Zika virus replicates in the maternal-fetal interface breaching the placental barrier and infecting the fetal tissues [27–29]. Zika virus crosses the placental barrier to reach the intrauterine cavity with the fetus and replicates in placental trophoblasts. Zika virus infection during all three trimesters of pregnancy may result in fetal infection and congenital abnormalities in newborns; however, severe clinical disease was often attributed to infections in early pregnancy [30]. While clinical outcomes of transplacental JEV infection are described in humans [31] and pigs [32], cellular targets and tissue tropism in the upper reproductive tract are unknown. To better understand JEV pathogenesis during transplacental infection, we studied JEV infection phenotypes in human primary endometrial cells and trophoblast. In pigs, we studied infection in the endometrium, placenta, and fetuses.

## Materials and methods

### Ethics statement

We followed the Canadian Council on Animal Care guidelines and Animal Use Protocol #20200106 approved by the University of Saskatchewan’s Animal Research Ethics Board and Animal Care and Use Review Office (ACURO) of US Army Medical Research and Development Command. All efforts were made to minimize animal suffering. Pigs were euthanized with an anesthetic overdose followed by exsanguination.

### Cells

C6/36 cells (ATCC, CRL-1660) were cultured in a Minimum essential medium (MEM; Sigma M4655) supplemented with 10% FBS and 1x Penicillin-streptomycin. VERO E6 cells (ATCC CRL-1586) were cultured in DMEM supplemented with 3% FBS, 1x Penicillin-streptomycin, and 2.67 mM Sodium bicarbonate (Gibco 25080–094). Human vaginal epithelial cells from two donors (ATCC, PCS-480-010 and Lifeline Cell Technology LLC, FC-0083) were cultured in a Vaginal epithelial cell basal medium (ATCC, PCS-480-030) with a Vaginal epithelial cell growth kit (ATCC, PCS-480-040). The commercial supplier confirmed the phenotype by staining cells with epithelium-specific (Pan-CK) and anti-fibroblasts (TE-7) antibodies. Human primary endometrial epithelial cells (Lifeline Cell Technology, FC-0078) were cultured in ReproLife female reproductive epithelial cell culture media with supplements (Lifeline Cell Technology; LL-0068). HTR-8/SVneo trophoblast cells (ATCC CRL-3271) were cultured in Roswell Park Memorial Institute 1640 Medium (RPMI; Gibco 11875119) supplemented with 5% FBS and 1x Penicillin-Streptomycin. VERO E6, human reproductive epithelial cells, and HTR-8/SVneo trophoblast were cultured at +37°C and C6/36 at +28°C in a 5% CO_2_ humidified incubator. All cells were mycoplasma free as confirmed by LookOut Mycoplasma PCR Detection Kit (Sigma-Aldrich).

### Viruses

The JEV Nakayama strain (GenBank EF571853) stock was initially produced at the World Reference Center for Emerging Viruses and Arboviruses, the University of Texas Medical Branch at Galveston, and transferred to our facility through the Public Health Agency of Canada. We inoculated VERO E6 cells and harvested media 9 days after inoculation to produce the working stock. Culture media containing JEV was centrifuged (12,000g, 20 min, +4°C); the supernatant was collected, aliquoted, and frozen at -80°C. The virus stock was mycoplasma free as confirmed by LookOut Mycoplasma PCR Detection Kit (Sigma-Aldrich).

### Reverse transcription-quantitative polymerase chain reaction (RT-qPCR)

We used QIAamp Viral RNA Mini Kit (QIAGEN) according to the manufacturer’s instructions to purify JEV RNA from 140 μl of virus stock, maternal and fetal blood plasma, maternal vaginal and nasal swabs, and the supernatants of different cell cultures. Maternal and fetal tissue samples were dissected and weighed on analytical balances. One ml of TRI Reagent Solution (Thermo Fisher Scientific) was added to 80–100 mg of tissues before homogenization (5 min at 25 Hz) with RNase-free stainless-steel beads and TissueLyser II (QIAGEN). Then, RNA extraction was performed with PhaseMaker tubes (Thermo Fisher Scientific) and PureLink RNA Mini Kit (Thermo Fisher Scientific) according to the manufacturer’s instructions.

For JEV RNA quantification, we used the previously described probe-based one-step RT-qPCR assay [33]. All RT-qPCR reactions were conducted on the StepOne Plus platform (Life Technologies, USA) and analyzed using StepOne software version 2.3. The reaction mixture (20 μl) for RT-qPCR (Bioline) consisted of 10 μl 2x SensiFAST Probe One-Step Mix, 0.4 μl RiboSafe RNase Inhibitor, 0.2 μl reverse transcriptase, 1 μl (500 nM) of forward (Universal-JEV-F: 5’-GCCACCCAGGAGGTCCTT-3’) and reverse (Universal-JEV-R: 5’- CCCCAAAACCGCAGGAAT-3’) primers, 0.5 μl (250 nM) probe (Universal-JEV-Probe: 56-FAM-CAAGAGGTG /***ZEN***/ GACGGCC-3***IABkFQ***), 1.9 μl nuclease-free water and 4 μl of sample RNA. A reverse transcription step of 10 min at 48°C and an enzyme activation step of 2 min at 95°C were followed by 40 amplification cycles (10 s at 95°C and 20 s at 60°C). RNA from a stock of JEV was used to generate standard curves that had a wide dynamic range (10^2.5^–10^12.5^ RNA copies/ml) with the high linear correlation R^2^ = 0.99 between the cycle threshold (Ct) value and template concentration. The standard curve was used to find the detection limit at Ct 40. Assay values were corrected for fluid volumes or tissue weights and upon logarithmical transformation expressed as JEV RNA genome copies per ml or gram.

Productive infection in tissues was confirmed with JEV negative-strand-specific RT-PCR: cDNA was synthesized with SuperScript III First-Strand Synthesis System (Invitrogen) using 10 pmole of the JEV-MinusStr forward primer 5-GGTCAGAACCACTACTGACAGT-3. Afterward, cDNA was amplified using the primers Universal-JEV-F and Universal-JEV-R (500 nM of each) and Universal-JEV-Probe (250 nM) described above. An enzyme activation step of 2 min at 95°C was followed by 60 amplification cycles (10 s at 95°C and 20 s at 60°C).

In all RNA extraction and PCR assays, we used VERO E6 cell culture media containing JEV as a positive PCR control. As a negative control, we used samples from non-manipulated control animals from our previous studies [34,35]. Strict precautions were taken to prevent PCR contamination. Aerosol-resistant filter pipette tips and disposable gloves were always used. Kit reagent controls were included in every RNA extraction and PCR run.

### Detection and quantification of infectious virus

We used the endpoint dilution assay in VERO E6 cells to isolate and quantify infectious titers in the JEV stock, blood plasma (maternal and fetal), nasal, and vaginal swabs (maternal) [34–39]. Fluids were serially diluted five-fold in four replicates starting from 1:10 in DMEM media (Thermo Fisher Scientific) supplemented with 5% FBS, a mixture of antibiotics (1,000 IU/ml penicillin and 1 mg/ml streptomycin, Gibco), and 2.25 g/l Sodium Bicarbonate (Thermo Fisher Scientific). Fifty μl of each dilution was added to confluent VERO E6 cells cultured in 96-well plates. After 2 hours of incubation at 37°C, 150 μl of fresh media was added to each well. The cells were incubated for seven days at 37°C. After washing and then drying for at least 4 h, the plates were kept at -20°C for at least 2 hours or until use. Anti-pan flavivirus E protein monoclonal antibodies clone D1-4G2-4-15 (ATCC; HB-112) [40] were used for immunohistochemistry staining [34–37,39] to detect JEV-infected cells. Fifty percent endpoint titers were calculated by the Spearman-Kärber formula and expressed in a decimal logarithm of a 50% infection dose for cell cultures (log_10_ TCID_50_) per ml. Media from mock-inoculated cells were used as negative controls.

Maternal blood, nasal swabs, and vaginal swabs which were negative or caused cytotoxic effects on VERO E6 cells, were used to inoculate C6/36 cells for virus isolation. Cells in 96-well plates were inoculated with undiluted or 1:10 diluted blood plasma or swabs in MEM media supplemented with 10% FBS, 1,000 IU/ml penicillin, 1 mg/ml streptomycin, 1x Gentamicin/Amphotericin Solution (Thermo Fisher Scientific), and 2.25 g/l Sodium Bicarbonate. After 12 hours of incubation at 37°C, fluids were removed, replaced with media, and cells were incubated for seven days at 28°C. Afterward, plates were fixed and stained with D1-4G2-4-15 antibodies as described above.

To make 10% suspension for titration, maternal tissues were homogenized in media with TissueLyser II (QIAGEN) for 3 min at 20 Hz. Samples were centrifuged at 2,000g for 10 minutes, and after two hours of incubation on C6/36 cells at 37°C, 150 μl of fresh media was added to each well, and cells were incubated for seven days at 28°C. Afterward, plates were fixed and stained with D1-4G2-4-15 antibodies as described above.

### *In vitro* infection phenotypes in human reproductive epithelial cells and trophoblast

We used human primary vaginal epithelial cells from two healthy deceased female donors: a 24-years-old African American donor (ATCC, PCS-480-010; Lot Number 80924222; 3^rd^ passage) and an 18-years-old Caucasian donor (Lifeline Cell Technology FC-0083; Lot Number 04033; 3^rd^ passage). Also, we used primary human endometrial epithelial cells from a healthy 13-years-old African American donor (Lifeline Cell Technology FC-0078; Lot Number 09953; 3rd passage). Information on the menstrual phase of human donors was not available, as the samples were de-identified. Cells were free for bacteria, yeast, fungi, mycoplasma, hepatitis B, hepatitis C, HIV-1, and HIV-2, as confirmed by manufacturers with sterility tests and PCR. In JEV studies, three technical replicates were included for cells from each biological donor. Twenty-five thousand vaginal or endometrial epithelial cells were seeded in 96-well plates in appropriate media. The next day, cell monolayers were inoculated with JEV at MOI of 0.1 or 10 in 100 μl of the same media. Plates were incubated at +37°C for 2 hours. Afterward, cells were washed three times with sterile PBS and covered with 200 μl media. Mock-infected cells were included as controls in each plate. Infected plates were incubated (5% CO_2_, +37°C) for 0, 3, 5, and 7 days when supernatants were collected, clarified (2,000 g, 5 min), and frozen (−80°C) for subsequent JEV load quantification. After the supernatant collection, plates with cell monolayer were dried for at least 4 hours and frozen (-80°C). Plates were stained with flavivirus-specific D1-4G2-4-15 antibodies as described above, and infected cells were visualized with a bright-field microscope. The same protocol was used to determine JEV infection kinetics in HTR-8/SVneo trophoblast cells (ATCC CRL-3271).

### RNA-seq and bioinformatics

Primary vaginal epithelial cells from two human donors were seeded into 24-well plates with 10^5^ cells per well. On the following day, cells in four wells representing technical replicates were inoculated with MOI 10 of JEV prediluted in DMEM media, and four wells were mock-inoculated with virus-free media from VERO E6 cells prediluted in the same way. At 48 hours after inoculation, cells were homogenized in 1 ml of TRI Reagent Solution (Thermo Fisher Scientific), and RNA was extracted according to the manufacturer’s protocol. RNA was assessed on a bioanalyzer and all samples had RNA Integrity Number (RIN) values above 8.0. DNA from samples was removed with TURBO DNA-free Kit (Thermo Fisher Scientific). mRNA with intact poly(A) tails were enriched with NEBNext Poly(A) mRNA Magnetic Isolation Module (New England Biolabs) and used for library constructions with NEBNext Ultra II Directional RNA Library Prep Kit for Illumina and NEBNext Multiplex Oligos for Illumina (96 Unique Dual Index Primer Pairs; New England Biolabs).

Libraries were sequenced on the NextSeq as paired-end reads using the NextSeq 500/550 High Output Kit v2.5 (150 cycles) (Illumina). FASTQ files were trimmed for adaptor sequences and filtered for low-quality reads using *Trimmomatic*. On average, 22.1 million reads per sample were generated. RNA-seq analysis was performed as we previously described [34]. Briefly, a complete transcriptome database was generated from ENSEMBL *Homo sapiens* GRCh38.p13 (GCA_000001405.28). Sequencing data were mapped and quantified using *kallisto* [41]. Then counts were analyzed using R *BioConductor* packages *tximport*, *edgeR* and *limma*. The *voom* function from the *limma* package was used for differential expression analysis. Gene set enrichment analysis was performed with *camera* function in *limma* using the GMT file (version 7.5.1) containing symbols of gene sets derived from the Gene Ontology Biological Process Ontology of the Gene Set Enrichment Analysis (GSEA) Molecular Signatures Database (MSigDB).

The set enrichment results from *camera* were graphed in *Cytoscape* using the *EnrichmentMap* plugin [34,42]. All networks were generated using a Jaccard + Overlap with a cutoff of 0.375 and a Combined Constant of 0.5. Sub-networks were discovered using GLay cluster and annotated using the WordCloud plugin of the top 4 words with a bonus of 8 for word co-occurrence. An accession number for RNA-seq data is PRJNA823367 in NCBI BioProject.

### Animal experiment

Eight female Landrace-cross pigs were purchased from the university high-health status herd free from porcine reproductive and respiratory virus (PRRSV), porcine parvovirus (PPV), congenital porcine circovirus 2 (PCV2), and porcine circovirus 3 (PCV3), which can cause fetal infection in pigs. Accordingly, maternal and fetal samples were negative for PRRSV, PPV, PCV2, and PCV3 in virus-specific PCR assays [35,43]. Before delivering to containment, six pigs were synchronized and bred with semen from a single donor to reduce biological variability; two pigs (G and H) remained non-pregnant. Insemination was scheduled to ensure that at the time of pig inoculation with JEV, three stages of pregnancy (the duration of pregnancy in pigs is 114 days) were represented: Two pigs at early pregnancy (inoculation at 30 days of pregnancy; pigs A and B), two pigs at mid-pregnancy (54 days; C and D), and two pigs at late pregnancy (86 days; E and F). The pregnancy was confirmed with ultrasound, and all pigs were delivered to Vaccine and Infectious Disease Organization, University of Saskatchewan biosafety level 3 containment facility. Animals were housed in two identical rooms in individual pens with no contact with each other. After seven days of acclimatization in containment, all pigs were sedated and inoculated with 10^7^ TCID_50_ of JEV intradermally (ear skin, 1 ml) + intravenous (ear vein, 1ml); this inoculation dose and routes were previously used for JEV inoculation in young piglets [8,44]. Clinical signs, including appetite, activity, and rectal temperature, were recorded before and after JEV inoculation.

We collected blood from the jugular vein with BD Vacutainer Plastic Blood Collection EDTA tubes; nasal and vaginal swabs were also collected. Samples were collected before JEV inoculation and at 1–7, 14, 21, and 28 days after virus inoculation. After blood centrifugation (2,000g, 20 min, +4°C), plasma was aliquoted and frozen at -80°C. For nasal and vaginal swabs, swabs were inserted into the top of the nose or vagina and rotated to obtain secretions. Afterward, a swab was placed into a tube containing 500 μl sterile media, the handle was broken one centimeter from the top of the swab, and the tube was stored at -80°C.

Pigs were euthanized and sampled 28 days after JEV inoculation. In all pigs, we sampled and froze maternal tonsils, mesenteric lymph nodes, brains, nasal mucosa, and vaginal mucosa with individual sterile instruments. In two non-pregnant pigs, uterine walls with endometrium were sampled and frozen. In six pregnant pigs, uteri with fetuses were removed to sample each fetus (14–15 fetuses per pig) with individual sterile instruments. First, a uterine wall with the placenta was collected from each conceptus (a fetus with fetal membranes) and frozen. Second, umbilical cord blood was aspirated from each fetus with sterile syringes and needles, centrifuged, and plasma was aliquoted and frozen at -80°C. Finally, fetuses were removed, inspected for gross pathology, and whole fetal brains were collected and frozen.

### Serology

We used an adapted virus-neutralizing assay to quantify JEV-neutralizing antibodies in maternal blood plasma [35,37]. Briefly, 50 μl of JEV (10^4^ TCID_50_/ml) were mixed with equal volumes of two-fold serially diluted plasma (in two replicates) and incubated at +37°C for 1 h before inoculation VERO E6 cells in 96-well plates. After 2 h, 100 μl/well of fresh DMEM supplemented with 1% FBS, 1x Penicillin-Streptomycin and 2.67 mM Sodium Bicarbonate was added. After 7 days, cells were fixed and stained with D1-4G2-4-15 antibodies as described for virus titration. The neutralizing antibody titers were calculated as the highest plasma dilution inhibited JEV infection in 50% of the inoculated wells.

We also quantified JEV-specific IgG antibodies in maternal blood plasma with immunoperoxidase monolayer assay (IPMA) [35,37]. Briefly, VERO E6 cells in 96-well cell culture plates were inoculated with 50 μl media containing 10^4^ TCID_50_/ml of JEV and incubated for 2 h (+37°C, 5% CO_2_). Then 100 μl of the culture medium (DMEM supplemented with 5% FCS, 1x Penicillin/Streptomycin, 2.67 mM Sodium Bicarbonate) was added. After 7 days of incubation at +37°C, 5% CO_2_ plates were dried for at least 4 hours and stored at -20°C until use. Plates with cells were thawed, dried, and fixed in 10% buffered formalin for 1 hour, and washed twice with 1x DPBS (pH 7.2). Afterward, fixed cells were incubated with 100% methanol in the presence of 0.3% H_2_O_2_ for 10 min. Then plates were washed with DPBS, and two-fold serial dilutions of tested blood plasma were added, followed by 1 hour incubation at +37°C. Plates were washed three times with DPBS containing 0.05% Tween-80 and 50 μl/well of rabbit anti-pig IgG (1:400, Abcam, ab136735) conjugated with horseradish peroxidase were added. After incubation for 1 hour at +37°C and washing, a color reaction was initiated by adding substrate solution: 1 mM 3-amino-9-ethylcarbazole, 5% N,N-dimethylformamide, 50 mM Sodium Acetate (pH 5.0), and 10 mM H_2_O_2_ (H_2_O_2_ was added just before placing on cells). The reaction was stopped by replacing the substrate with an acetate buffer, and JEV-specific staining was determined by examination with a microscope. The titers were defined as the log reciprocal of the highest serum dilution. Blood plasma samples of mock-inoculated control animals from our previous studies were used as a negative control.

### Interferon-alpha quantification

To quantify interferon-alpha (IFN-α), maternal and fetal blood plasma samples were diluted 1:2 and tested with Invitrogen Porcine IFN-alpha ELISA Kit (Thermo Fisher Scientific) according to the manufacturer’s instructions.

### Immunohistochemistry

We used Mouse and Rabbit Specific HRP/DAB IHC Detection Kit—Micro-polymer (Abcam, ab236466) to identify JEV antigen in fetal brains. Fetal brain cryosections of 10 μm were fixed in 10% buffered formalin for 15 min at +4°C. After treatment with 0.3% H_2_O_2_ and 1% Triton X-100 for 15 min and protein block for 10 minutes, tissue sections were washed in PBS and incubated with mouse monoclonal antibodies D1-4G2-4-15 (1:10) against flavivirus E protein for 1 hour at +37°C. Afterward, the sections were washed and incubated with mouse specifying reagent, goat anti-rabbit HRP-conjugate, DAB chromogen, and DAB substrate according to the kit’s instructions. Subsequently, tissues were counterstained with hematoxylin and analyzed with a light microscope.

For JEV antigen identification in human reproductive epithelial cells and trophoblast, cells were fixed and stained with D1-4G2-4-15 antibodies as described above in the detection and quantification of infectious JEV.

### Statistical analysis

We used GraphPad PRISM 8 software to analyze data. The difference with p < 0.05 was considered statistically significant. Viral loads and immunology data were expressed as individual values and mean ± standard deviation (M ± SD). Japanese encephalitis virus RNA loads and infectious titers in fetal blood plasma collected at euthanasia were compared between fetuses from early, mid, and late pregnancy subgroups using one-way ANOVA; Tukey’s multiple comparison test was used for multiple comparisons between fetuses of different subgroups. The same statistical tests were used to compare JEV RNA loads in the placenta and fetal brains. The number of fetuses with pathology was compared between subgroups with Yates-corrected χ2-test. We used the Spearman correlation to evaluate relationships between fetal IFN-α levels in blood plasma and JEV loads.

## Results

### Japanese encephalitis virus causes persistent infection and molecular pathology in human vaginal epithelial cells

It is unknown whether JEV has tropism in the vaginal epithelium and the potential for sexual transmission in humans. Here, we assessed virus infection kinetics in human vaginal epithelial cells and transcriptional responses to infection.

**Fig 1:**
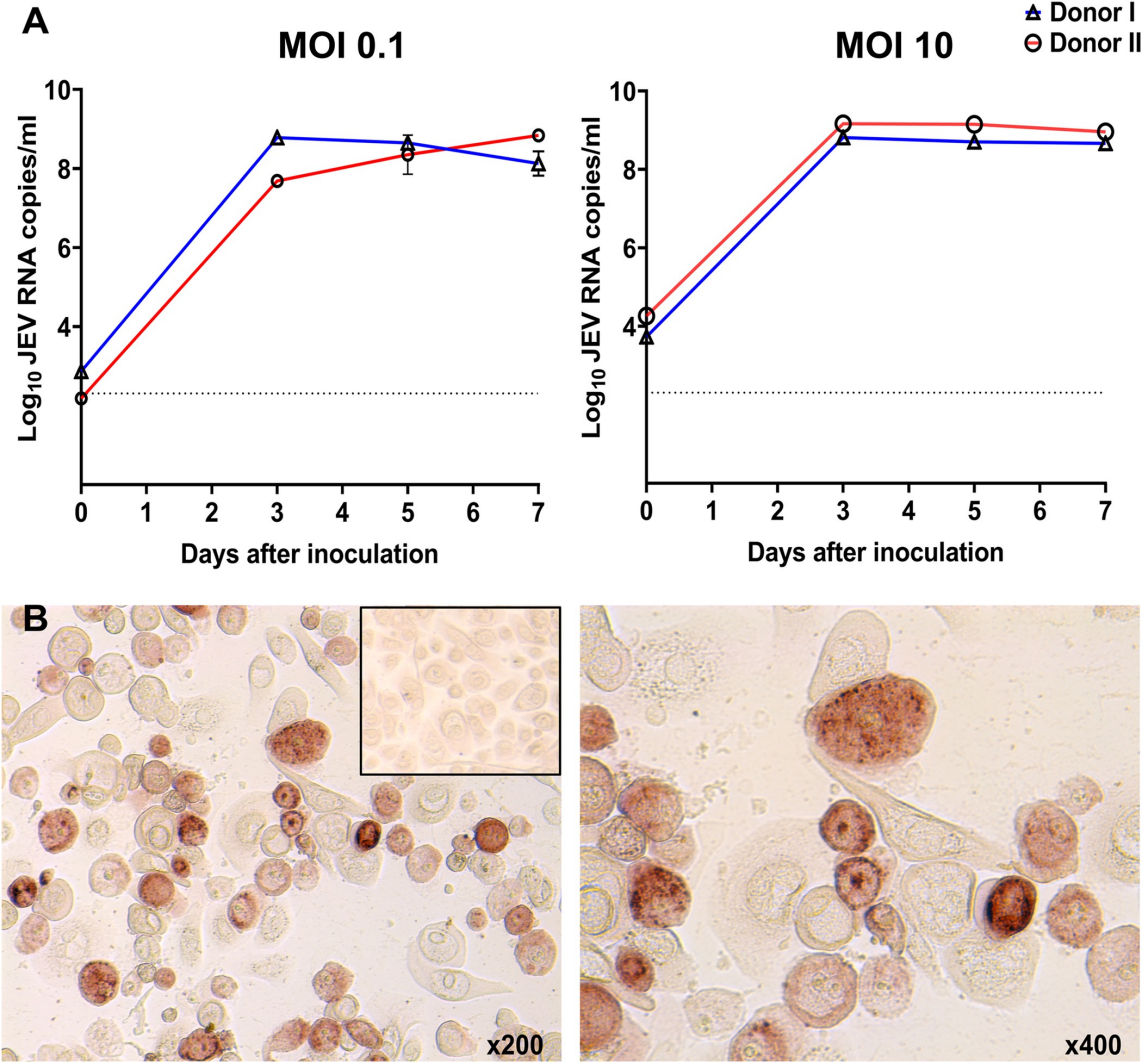
Japanese encephalitis virus causes persistent infection in human primary vaginal epithelial cells. (**A**) JEV infection kinetics in human primary vaginal epithelial cells. (**B**) Immunohistochemistry specific for the JEV envelope protein in human vaginal epithelial cells at 7 days after inoculation (MOI 10). The combination of virus-positive and virus-negative cells in close proximity was observed. The insert shows mock-inoculated cells.

We inoculated primary vaginal epithelial cells from two donors and quantified viral loads in the cell culture supernatant on different days. The virus caused persistent infection with very high loads at 3, 5, and 7 days after inoculation (**Fig 1A**). The virus loads were similar in the vaginal epithelium of two donors and at different MOIs (**Fig 1A**). The productive persistent infection, at least for 7 days, was confirmed by immunohistochemistry specific for the JEV envelope protein (**Figs 1B** and **S1**). Interestingly, we observed the combination of virus-positive and virus-negative cells in close proximity at each sampling time (**Fig 1B**).

**Fig 2:**
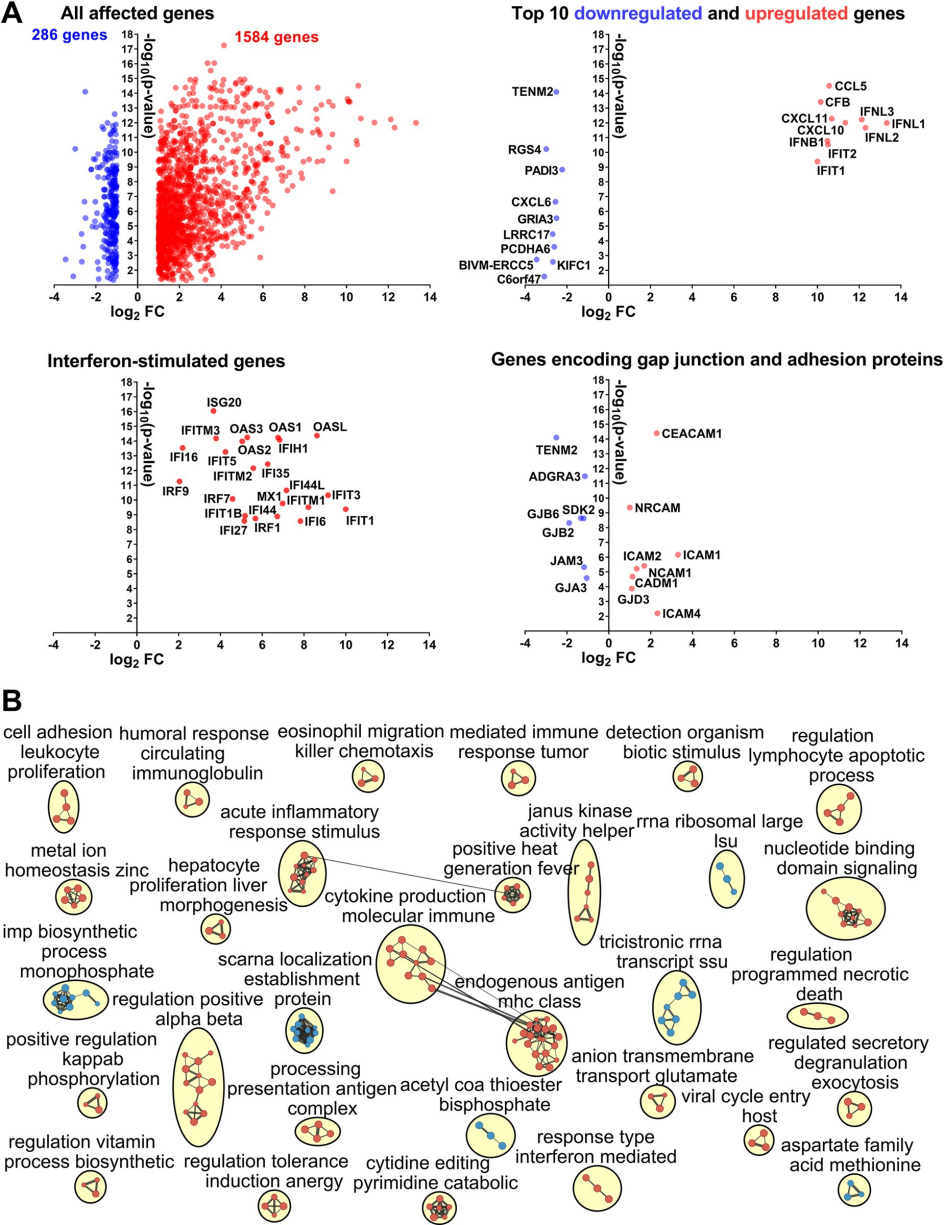
Cellular transcriptional responses during JEV infection in human primary vaginal epithelial cells. (**A**) Cellular transcriptional responses in human primary vaginal epithelial cells during JEV infection. Plots of the upregulated (red) and downregulated (blue) genes. All affected genes, top 10 affected genes, interferon-stimulated genes, and genes encoding gap junction and adhesion proteins with FDR-adjusted p < 0.05 and log2 fold change (FC) > 1 are shown. See raw data in **S1A Table**. (**B**) Molecular pathology network in human primary vaginal epithelial cells during JEV infection. An enrichment map of significantly altered GO biological processes is shown. Red are pathways with positive and blue are with negative enrichment. All subnetworks with FDR-adjusted *p* < 0.05 and at least three connected nodes are shown. See raw data in **S1B Table**.

Next, to better understand molecular pathology induced by JEV infection in the vaginal epithelium, we quantified whole-genome expression in vaginal epithelial cells. Gene expression differed considerably between control and JEV-infected cells with 1584 upregulated and 286 downregulated genes (FDR-adjusted p < 0.05; log_2_ fold change (FC) > 1; **Fig 2A**, **S1A Table**). Among the top ten upregulated genes with 10–13 log_2_ fold change were genes encoding interferon lambda 1, 2, 3 and interferon beta 1 (IFNL1, 2, 3 and IFNB1; **Fig 2A**). In accordance with previous findings where treatment of human vaginal epithelial cells with interferon lambda and beta induced a strong interferon-stimulated gene (ISG) response [18], we also found overexpression of 23 canonical ISGs (**Fig 2A**). In addition, among the top ten upregulated genes were genes encoding proinflammatory chemokines CCL5, CXCL10, and CXCL11, which recruit leukocytes to the site of inflammation [45,46]. Fifteen genes encoding gap junction and adhesion proteins were also affected (**Fig 2A**), suggesting changes related to epithelial barrier function. Accordingly, enrichment of Gene Ontology (GO) biological processes showed significant effects in the JEV-infected epithelium with 255 upregulated and 78 downregulated processes (FDR-adjusted p < 0.05; **Fig 2B; S1B Table**). Specifically, genes with altered expression were positively enriched for processes related to type I, II, and III interferon responses (12 GO processes FDR-adjusted p < 0.05), inflammatory responses (10 GO processes), and virus-host interactions (5 GO processes) (**Fig 2B**; highlighted in **S1B Table**). Interestingly, “negative regulation of morphogenesis of an epithelium” and “negative regulation of bicellular tight junction assembly” GO processes were also enriched (**S1B Table**).

In accordance with infection kinetics and molecular pathology, JEV caused cytopathology in vaginal epithelial cells with more prominent cell rounding and detachment compared to control cells (**S2 Fig**). Cell death started at 3 days after inoculation and aggravated at 5 and 7 days, and was dose-dependent. In conformity with immunohistochemistry where the combination of virus-positive and virus-negative cells was observed (**Fig 1B**), the combination of dead and live cells was also observed even at 7 days after inoculation (**S2 Fig**).

In summary, we discovered that JEV causes persistent infection in the human vaginal epithelium and has a potential for sexual transmission in humans. Also, RNA-seq analysis in human vaginal epithelial cells infected with JEV provides evidence for molecular pathology that leads to inflammation and disruption of epithelial barrier function. In support, the vaginal epithelium showed progressive cytopathology during JEV infection.

### Japanese encephalitis virus causes persistent infection and cytopathology in human endometrial epithelial cells and trophoblast

While clinical outcomes of transplacental JEV infection are described in humans [31], cellular targets in the upper reproductive tract are unknown. To better understand JEV pathogenesis during transplacental infection, we assessed virus infection kinetics in human endometrial epithelial cells and trophoblast.

**Fig 3:**
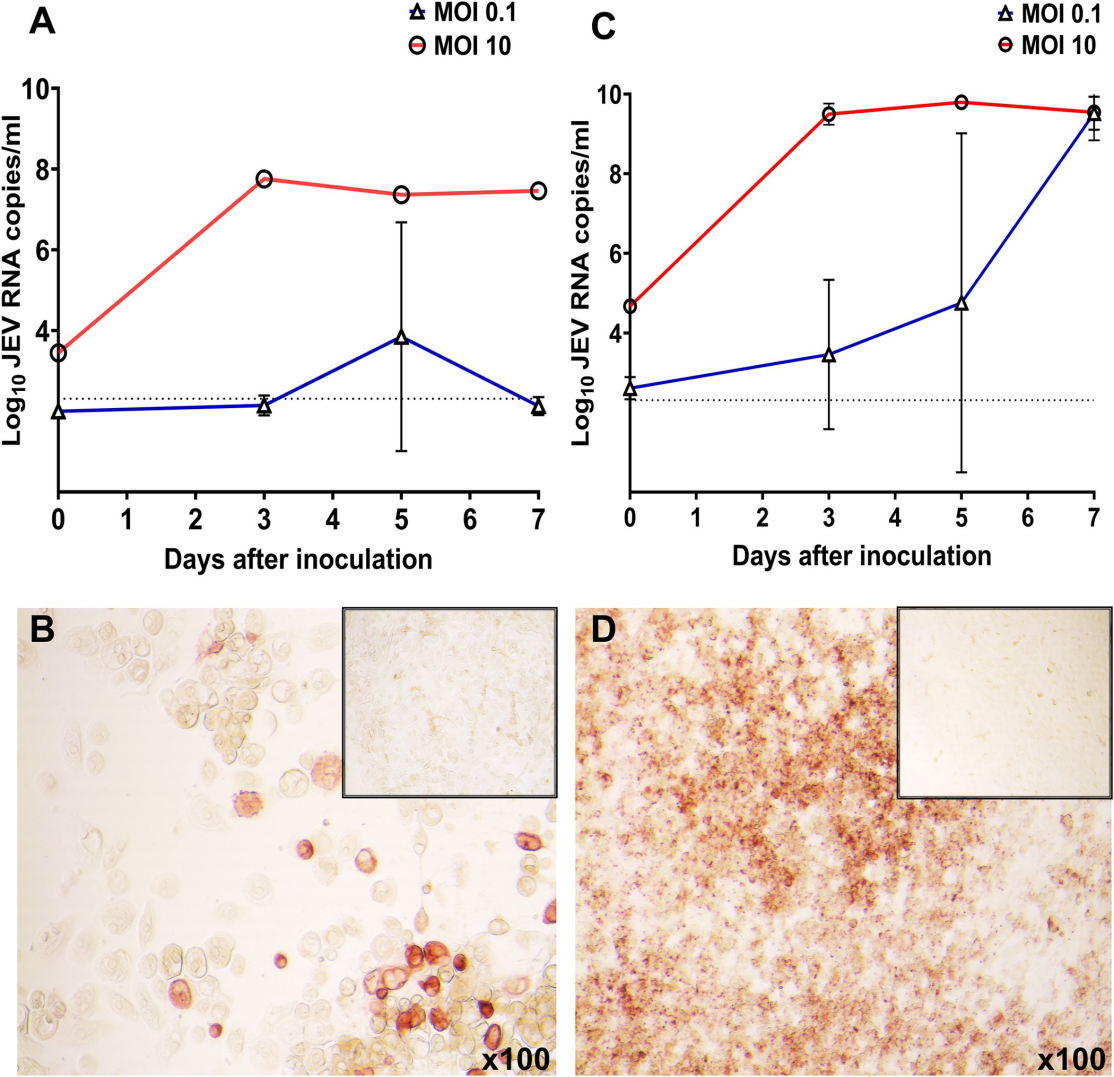
Japanese encephalitis virus causes persistent infection in human primary endometrial epithelial cells and trophoblast. (**A**) JEV infection kinetics in human primary endometrial epithelial cells. (**B**) Immunohistochemistry specific for the JEV envelope protein in human endometrial epithelial cells at 3 days after inoculation (MOI 10). The insert shows mock-inoculated cells. (**C**) JEV infection kinetics in human trophoblast—HTR-8/SVneo cells. (**D**) Immunohistochemistry specific for the JEV envelope protein in human trophoblast at 7 days after inoculation (MOI 10). The insert shows mock-inoculated cells. The dotted line on graphs represents the limit of detection.

In primary endometrial epithelial cells, JEV kinetics depended on the initial inoculation dose. At MOI 0.1, high viral loads in the supernatant and virus-positive cells were detected only in one well replicate at 5 days after inoculation (**Fig 3A**). In contrast, at MOI 10 high viral loads and virus-positive cells were detected from 3 to 7 days (**Figs 3A and 3B and S3**). We did not observe distinct cytopathology in infected cells; both control and infected epithelium had detached rounded cells (**S4 Fig**).

In human trophoblast, JEV kinetics also depended on the initial inoculation dose. At MOI 0.1, JEV loads in supernatants gradually increased from 3 to 7 days after inoculation (**Fig 3C**). At MOI 10 high viral loads were detected at 3, 5, and 7 days (**Fig 3C**). Immunohistochemistry showed highly intensive staining with many virus-positive cells on all sampling days for MOI 0.1 and 10 (**Figs 3D and S5**). In cells inoculated with MOI 10, gradually aggravating cytopathology with focal and diffuse cell detachment and disruption of the cell monolayer was observed from 3 to 7 days after inoculation. However, at MOI 0.1, we did not observe distinct cytopathology (**S6 Fig**).

In summary, we discovered that JEV causes persistent infection and cytopathology in the human endometrial epithelium and trophoblast, possibly contributing to transplacental JEV transmission.

### Japanese encephalitis virus persists in the lower reproductive tract of the native amplifying host

To further explore the potential of JEV for sexual transmission, we studied virus persistence and shedding in vaginal tissues of the native amplifying host—pigs.

**Fig 4:**
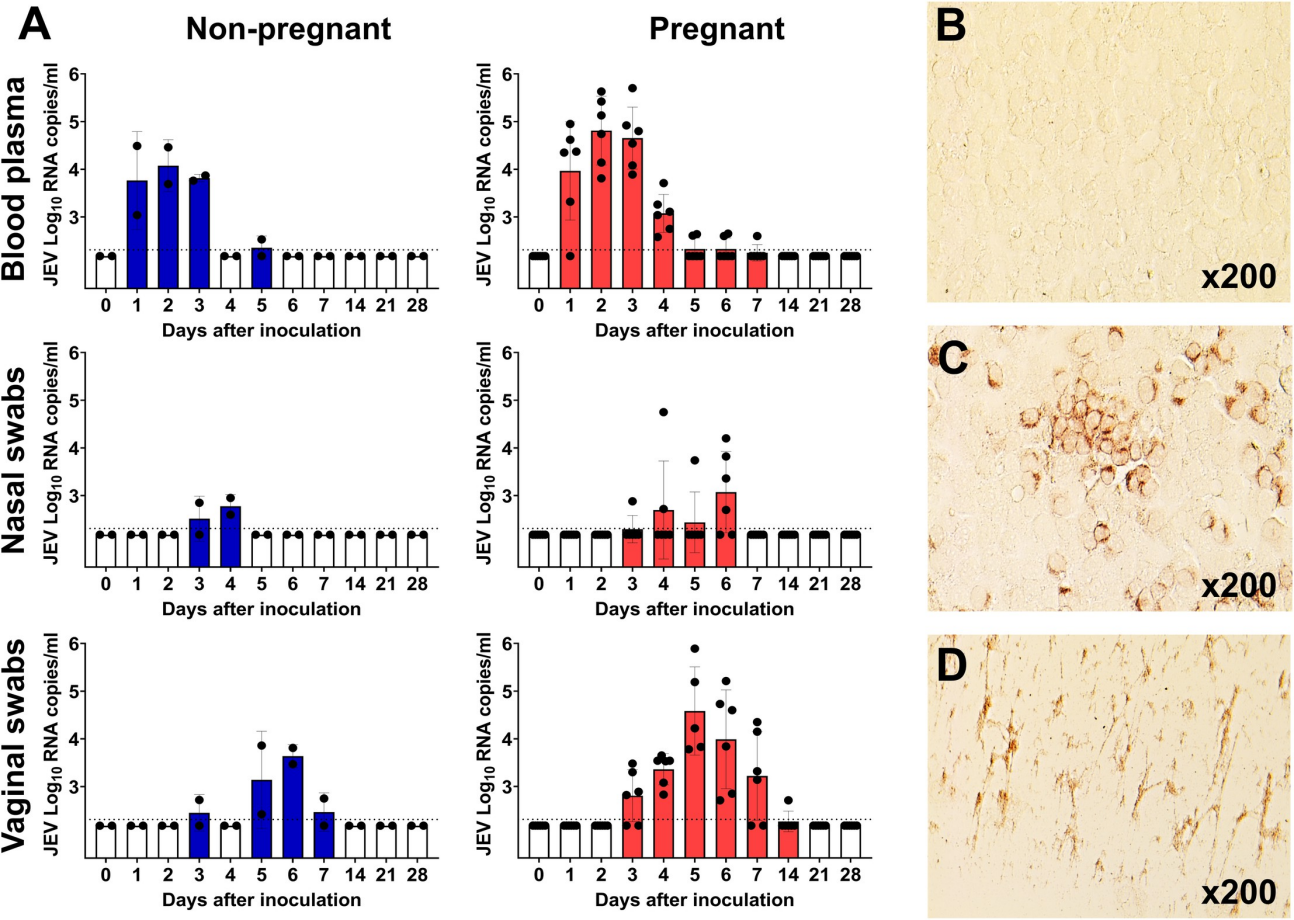
Japanese encephalitis virus loads in blood, nasal secretions, and vaginal secretions in non-pregnant and pregnant pigs. (**A**) JEV RNA loads determined by virus-specific RT-qPCR in blood plasma, nasal swabs, and vaginal swabs. The dotted line is the detection limit. Columns represent mean values with standard deviations. (**B**) VERO E6 cells inoculated with blood plasma collected from a pig before JEV infection. (**C**) JEV-positive staining (red) in VERO E6 cells inoculated with blood plasma collected from a mid pregnancy pig two days after JEV inoculation. In accordance with the highest viral RNA loads identified in pigs from the mid pregnancy subgroup at 2–3 days after inoculation, we isolated infectious JEV in VERO cells at the same time points. **(D)** Cytotoxicity in VERO E6 cells inoculated with maternal blood plasma. Isolation and titration of infectious flaviviruses from samples of immunocompetent mammalians is challenging, most probably because of insufficient sensitivity of cellular assays [19,21,39,47–49]. Most PCR-positive plasma samples did not show infectious JEV, indicating infectious titers below the detection limit of the VERO-based endpoint dilution assay. In addition, maternal blood plasma samples, even after 1:10 dilution, caused cytotoxicity. Similarly, inoculation of mosquito C6/36 cells, which can be more sensitive than VERO cells for flavivirus isolation [37], with undiluted or 1:10 diluted blood plasma led to strong cytotoxicity preventing interpretation of results. Nasal and vaginal swabs with initial sampling dilution caused cytotoxicity in VERO and C6/36 cells that halted the assay, and with subsequent higher dilutions, we did not isolate infectious JEV.

One non-pregnant pig showed elevated body temperature 39.3–39.4°C (the baseline temperature was 38.4–38.5°C) and decreased appetite at 3–4 days after JEV inoculation. Other animals did not show clinical signs. As previously demonstrated in young piglets [8], adult pigs also had JEV viremia (**Fig 4A–4C**), shedding in nasal secretions (**Fig 4A**), and virus-specific antibody responses (**S7 Fig**). As a host response to infection, during viremia and JEV shedding in body fluids, pigs showed decreased IFN-α concentrations (**S7 Fig**). Afterward, on days 14, 21, and 28, during JEV clearance in blood and body fluids, IFN-α concentrations were close to or exceeded the initial base concentrations.

Interestingly, in contrast to a previous study in young piglets [8], we discovered that adult pigs shed JEV RNA in vaginal secretions. Non-pregnant pigs shed JEV within 3–7 days after inoculation except on day 4. Pregnant pigs continually shed virus RNA within 3–7 days, and even at 14 days after inoculation (**Fig 4A**). Virus loads in non-pregnant pigs peaked at 4 Log_10_ RNA copies/ml. In pregnant pigs, JEV loads peaked at 6 Log_10_ RNA copies/ml (**Fig 4A**). Vaginal mucosa sampled at 28 days after inoculation was also positive for JEV RNA in two pigs (**S8 Fig**).

Altogether, we confirmed previous findings where pigs had JEV viremia and shedding in nasal secretions [8] and discovered that pigs shed JEV in vaginal secretions. Viral RNA was detected in vaginal secretions 2–10 days after it was cleared from blood plasma suggesting local and prolonged JEV replication in vaginal tissues.

### Japanese encephalitis virus persists in the upper reproductive tract of the native amplifying host and causes transplacental infection

To better understand the pathogenesis of JEV transplacental transmission, we studied infection in the endometrium, placenta, and fetuses of the native amplifying host—pigs.

As previously demonstrated in young piglets [8,50], adult pigs also had JEV persistence in tonsils, lymph nodes, brain, and nasal mucosa collected 28 days after inoculation (**S8 Fig**).

**Fig 5:**
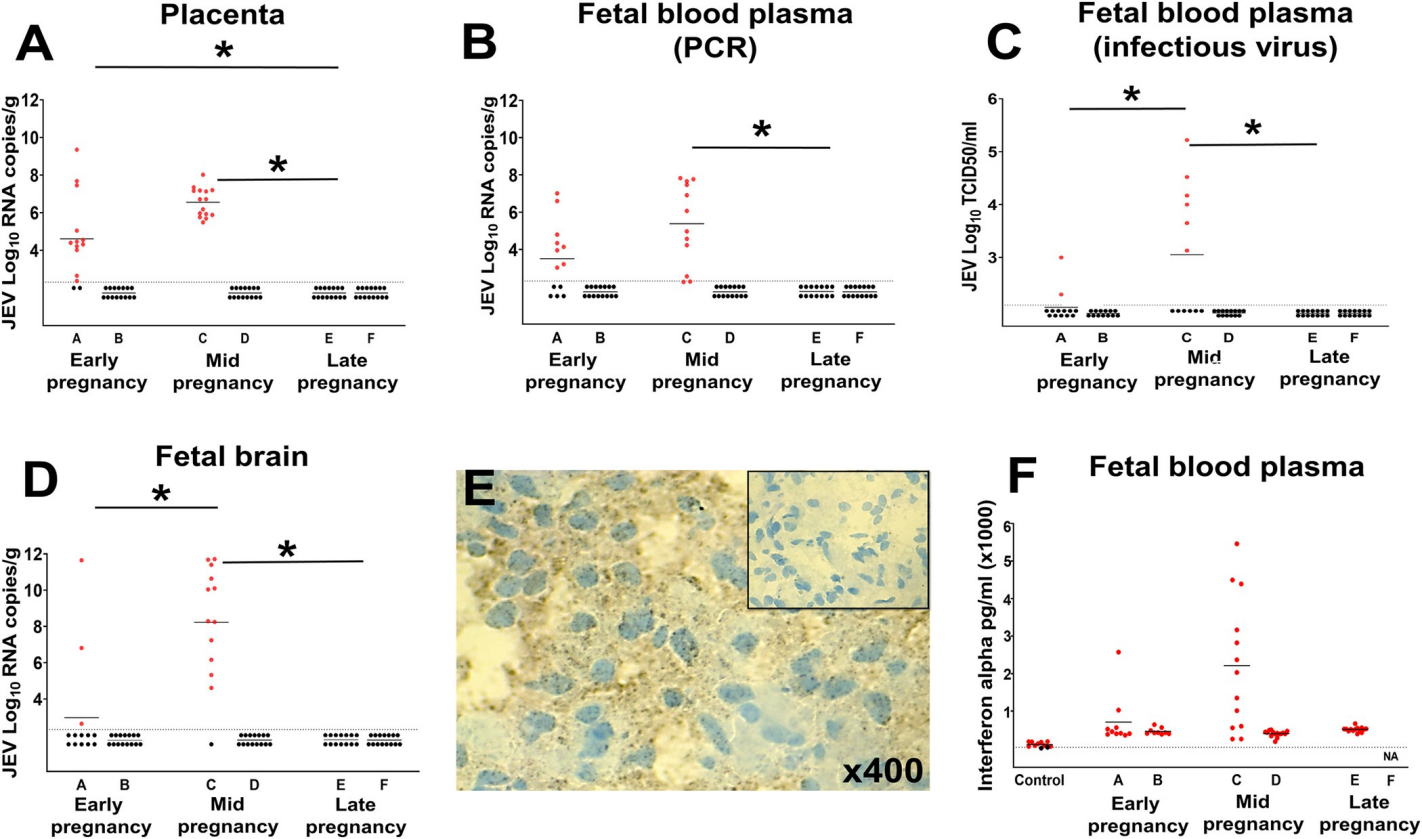
Japanese encephalitis virus infection in fetuses. (**A**) JEV RNA loads in the placenta. JEV RNA loads (**B**) and infectious titers (**C**) in fetal blood. (**D**) JEV RNA loads in fetal brains. (**E**) JEV-specific immunohistochemistry in the brain from a virus-negative fetus (insert) and in the brain from the fetus positive for JEV; brown staining represents cells positive for the JEV envelope protein. (**F**) IFN-α concentrations in fetal blood plasma. Control blood plasma samples were from fetuses of two healthy pigs sampled at 78 days of pregnancy in our previous studies [35]. Dots represent individual pigs or fetuses. The dotted line is the detection limit—36 pg/ml. In fetuses, the short horizontal line represents mean values. In all graphs, dots represent individual fetuses. The dotted line is the detection limit. The short horizontal line represents mean values. *: JEV loads were significantly different.

We also identified JEV persistence in one endometrial tissue sample out of two tested (**S8 Fig**). Virus persistence in the endometrium may lead to infection in the adjacent placenta and afterward in fetuses. Indeed, we found high JEV RNA loads in the placenta from nearly all (pig A, early pregnancy) or all (pig C, mid pregnancy) fetuses (**Fig 5A**). Accordingly, fetuses from pig A and pig C had high JEV RNA loads in their blood plasma as determined by PCR (**Fig 5B**). Two fetuses from pig A and six from pig C also had high infectious JEV titers in blood plasma as determined by virus isolation and titration (**Fig 5C**). Infectious JEV titers were significantly higher in fetuses from pig C inoculated at mid pregnancy than from pig A (p = 0.01) inoculated at early pregnancy (**Fig 5C**). Only one pregnant pig (50%) in early and mid pregnancy subgroups showed transplacental and fetal infections suggesting the stochastic nature of JEV transmission from mother to fetuses or unknown factors that may affect the virus spread through the placenta.

Fetuses from pigs A and C also had JEV RNA in their brains (**Fig 5D**). Three fetuses from pig A inoculated at early pregnancy had JEV RNA in their brains. Twelve out of thirteen fetuses (92.3%) from pig C inoculated at mid pregnancy had JEV RNA in their brains with high titers ranging from 4.6 to 11.7 Log10 RNA copies/g (**Fig 5D**). Virus RNA loads in brains were significantly higher (p = 0.001) in fetuses from pig C inoculated at mid pregnancy than in fetuses from pig A inoculated at early pregnancy. We also sectioned selected frozen fetal brains from pig A (early pregnancy) and pig C (mid pregnancy), and stained tissues with antibodies specific to the JEV envelope protein. In accordance with extremely high viral loads in the brain of fetus #10 (11.7 Log_10_ RNA copies/g) from pig A, its brain contained intensive diffuse JEV-specific staining (**Fig 5E**). However, we did not find JEV antigen in the brain of fetus #1 that had almost twice lower JEV loads (6.8 Log_10_ RNA copies/g), suggesting more focal antigen localization or insufficient sensitivity of our immunohistochemistry assay. Fetuses #6 (11.7 Log_10_ RNA copies/g) and #14 (11.4 Log_10_ RNA copies/g) from pig C also had intensive JEV-specific staining in brain tissues.

**Fig 6:**
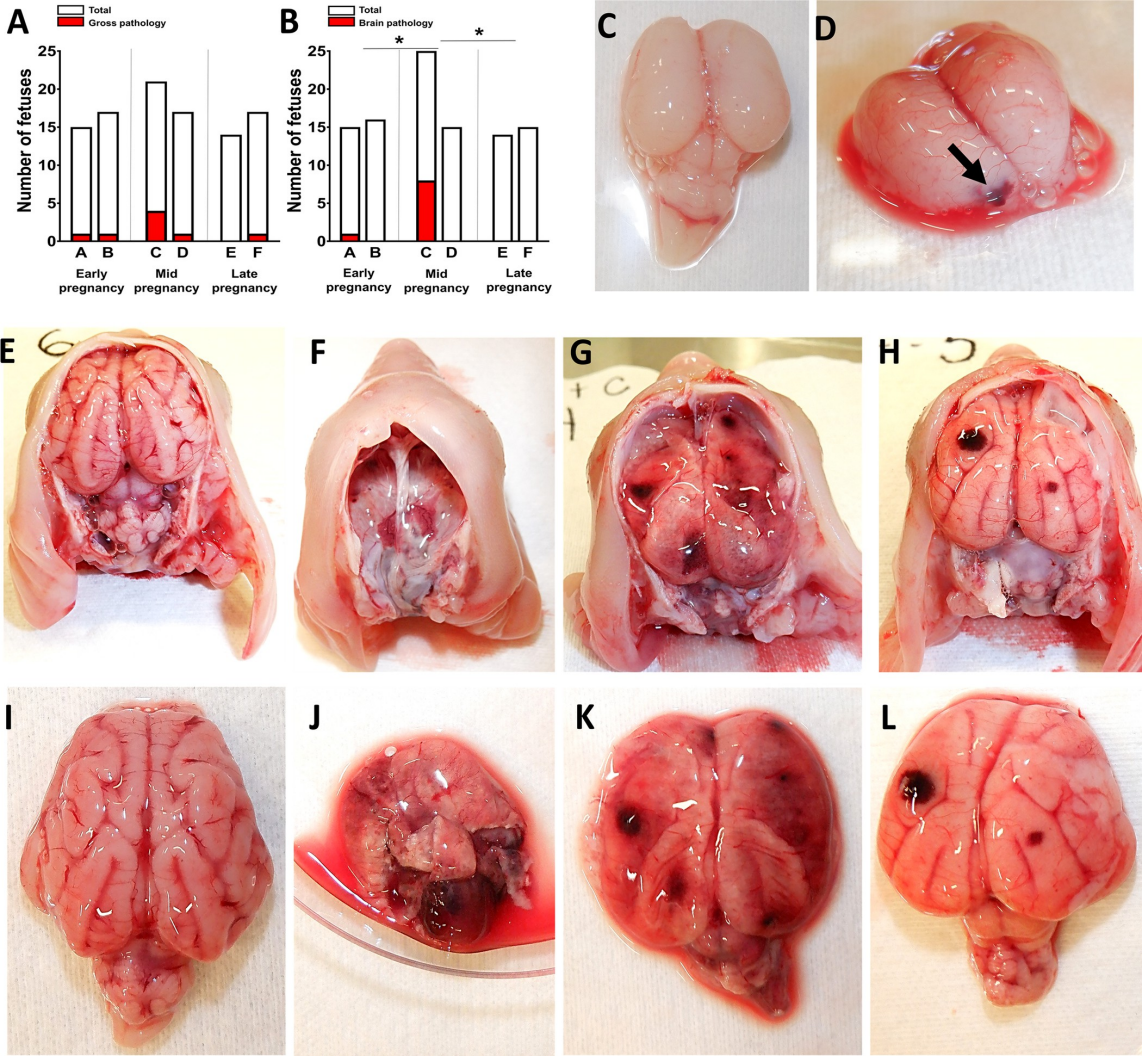
Fetal pathology. (**A**) The number of fetuses with gross pathology. (**B**) The number of fetuses with brain pathology. *: The number of fetuses with brain lesions was significantly higher in the mid pregnancy subgroup than in early pregnancy (p < 0.016) and late pregnancy (p < 0.0039) subgroups. The fetal brain with no lesions (**C**) and focal hemorrhage (**D**) from early pregnancy pig A. Mid pregnancy pig C: The fetal brain with no lesions (**E** and **I**). (**F**) The fetal skull with the completely autolyzed brain. (**J**) The fetal brain with complete autolysis. (**G** and **K**) The fetal brain with partial autolysis and diffuse hemorrhages. (**H** and **L**) The fetal brain with focal hemorrhages and less defined sulcus and gyrus structures.

In accordance with JEV infection in fetuses, pigs A and B had one fetus with pathology (7.1% and 6.3% of the total number of fetuses) (**Fig 6A**); specifically, fetuses had edema and hemorrhages (**S9 Fig**). Pigs C and D inoculated at mid pregnancy had four and one fetuses (23.5% and 6.3%) with edema, tissue autolysis, and mummification (**Figs 6A and S9**). Pigs E and F sampled at late pregnancy did not have fetuses with visible pathology; only one (6.3%) mummified fetus was found in pig F.

A fetus from pig A inoculated at early pregnancy, and many fetuses from pig C inoculated at mid pregnancy had brain lesions (**Fig 6B**). Fetuses with and without gross pathology had brain lesions (**S2 Table**), suggesting the fetal brain is a primary and vulnerable target for JEV. The fetus from pig A (early pregnancy) had focal brain hemorrhage (**Fig 6C and 6D**). Eight fetuses from pig C (mid pregnancy) had complete brain autolysis, partial brain autolysis, diffuse brain hemorrhages, and focal brain hemorrhages with less defined sulcus and gyrus structures (**Fig 6E–6L**). Fetuses from other pigs did not have brain lesions. The number of fetuses with brain lesions was significantly higher in the mid pregnancy subgroup than in early pregnancy (p < 0.016) and late pregnancy (p < 0.0039) subgroups.

Previous studies from our and other groups suggest that type I interferon *in utero* responses contribute to fetal pathology and death during congenital viral infections [34,38,39,51]. In the present study, two JEV-positive fetuses from early pregnancy pig A showed increased IFN-α levels (**Fig 5F**). In mid pregnancy, nine out of 13 fetuses of pig C showed increased IFN-α levels (**Fig 5F**); accordingly, interferon levels in blood plasma positively correlated with JEV loads in blood plasma (p = 0.019, r = 0.68).

Collectively, we showed that JEV persists in the endometrium for at least 28 days. Also, we discovered high virus loads in the placenta that allowed the virus to cross the placental barrier causing severe lesions in the fetal brain, fetal interferon-mediated immunopathology, and fetal death.

## Discussion

In the present study, we questioned the potential of JEV for sexual transmission. We also studied infection in endometrial and placental cells to better understand the pathogenesis of transplacental JEV transmission. For sexual transmission, we discovered that JEV causes persistent infection in the human vaginal epithelium leading to inflammation and disruption of epithelial barrier function. And that pigs—the native amplifying host for JEV—shed the virus in vaginal secretions. For transplacental transmission, we discovered that JEV causes persistent infection and cytopathology in the human endometrial epithelium and trophoblast. And that JEV persists in the endometrium and placenta of pigs with subsequent transplacental infection and fetal death.

There are emerging concerns about sexual flavivirus transmission. These concerns emerged during the recent Zika epidemic where many sexual transmissions in humans have been reported including male-to-male, male-to-female, and female-to-male transmissions [13–15]. Zika virus has been detected in vaginal secretions [24–26] replicating in human vaginal epithelial cells [18]. However, it was unknown whether JEV—another flavivirus related to Zika virus, also has tropism in the human reproductive epithelium and the potential for sexual transmission. This knowledge is important because almost a half of the world’s population lives in territories where JEV is permanently circulating with estimated 68,000 cases reported annually [4]. Because many JEV-infected humans do not develop apparent clinical signs, sexual JEV transmission may be underestimated. Similarly, Zika virus sexual transmission in sustaining the virus in the human population during the epidemic was most probably underestimated [52].

We showed that JEV causes persistent infection in the vaginal epithelium with high loads for at least 7 days (**Fig 1**). Transcriptional responses identified in human vaginal epithelial cells during JEV infection showed inflammation, altered bicellular junctions, and disruption of epithelial barrier function (**Fig 2**). The vaginal epithelium is the first line of defense against sexually transmitted infections in women and the disrupted epithelial barrier may facilitate the sexual transmission of JEV as well as other pathogens, for example human immunodeficiency virus 1 and chlamydia. High JEV titers and intensive antigen staining in infected cells persisted for at least 7 days despite type I and III interferon transcriptional responses, including interferon lambda—an antiviral cytokine that functions at barrier surfaces [18,53,54]. Interestingly, at both low (MOI 0.1) and high (MOI 10) inoculation doses and at each sampling time point, we observed many virus-positive cells with intensive red staining and virus-negative cells tightly adjacent to each other (**Figs 1B and S1**). We hypothesize that antiviral responses in JEV-infected epithelial cells will differ from responses in uninfected bystander cells. Thus, it will be interesting to assess transcriptional responses in infected and virus-free bystander epithelial cells to identify reasons for such phenotypical heterogeneity and better understand mechanisms employed by flaviviruses to subvert epithelial immunity.

Next, we discovered that non-pregnant and pregnant pigs shed JEV in vaginal secretions (**Fig 4**). Also, we found virus persistence in vaginal mucosa for at least 28 days (**S8 Fig**). These findings are unexpected because in a previous study the authors did not find JEV in vaginal secretions [8]. But previously, vaginal swabs were collected in young 7-week-old piglets before sexual maturity (pigs reach sexual maturity at 20–24 weeks). Here, JEV-inoculated pigs were on average 35-week-old. Thus, age and probably age-dependent hormonal changes determine JEV tropism in vaginal tissues and vaginal shedding. In support, hormonal changes in mice and non-human primates affect Zika virus replication in the female reproductive tract and transgenital transmission to internal organs [17–19,22].

Interestingly, infected pigs had considerably longer JEV shedding with higher titers in vaginal secretions than in nasal secretions (**Fig 4**) supporting the previous suggestion that the dampened innate response to RNA viruses in the lower female reproductive tract is an exceptional feature of the vaginal mucosa which may not extend to other mucosal surfaces [55].

Vaginal JEV shedding and persistence in vaginal mucosa were detected 2–10 and 21–23 days after the virus was cleared from blood plasma, suggesting local JEV replication in reproductive tissues (**Fig 4**). Isolation and titration of infectious flaviviruses from samples of immunocompetent mammalians is challenging [39,47–49], and similar to the previous Zika studies in non-human primates [19,21], we did not recover infectious JEV in vaginal tissues. The significance of the JEV RNA that persists in vaginal tissues with shedding after it is cleared from blood plasma needs to be further assessed. Interestingly, it has been reported that Zika virus preferentially replicates in the female reproductive tract of non-human primates after vaginal inoculation but not after peripheral inoculation [19]. Our data from the present study with peripheral inoculation and a previous study where pigs showed systemic infection after artificial insemination with virus-positive semen [56] suggest that JEV has strong tropism to female reproductive tissues independently of the route of infection.

Our study also contributed new knowledge for JEV pathogenesis during transplacental infection. We discovered dose-dependent JEV infection in the primary human endometrial epithelium (**Fig 3A and 3B**). The endometrial epithelium is the vital maternal point of first contact for an embryo during implantation and early development [57,58]. Thus, it will be important to identify whether silent JEV infection may impact interactions between embryo and endometrial epithelial cells and evoke early embryonic loss. Also, JEV persistence in the endometrium may lead to infection in the adjacent placenta and afterward in fetuses. Indeed, we discovered aggressive JEV infection and cytopathology in the trophoblast (**Figs 3C and 3D and S5 and S6**). The trophoblast is a specialized epithelium vital for placental function in supporting fetal development [59]. Virus replication in the trophoblast is the primary step toward transplacental and fetal infection. Human HTR-8/SVeno cells used in the present study were derived from chorionic villi explants of the human first-trimester placenta. Aggressive JEV infection in the early HTR-8/SVneo trophoblast supports previous clinical findings in humans where JEV infection in at least five pregnant women was described during an extensive outbreak in India. And abortions occurred after infection with JEV early in pregnancy, while newborns of women infected near term did not show obvious congenital pathology [31].

Supporting the role of the pregnancy stage in infection phenotypes in reproductive tissues, JEV crossed the placental barrier in pigs in only early and mid pregnancy (**Fig 5**) and did not cause placental and fetal infections in late pregnancy. Our findings of JEV infection in the porcine placenta and fetal brains at early pregnancy are consistent with a historical study where pigs infected at early pregnancy delivered stillborn or abnormal piglets [32]. However, transplacental infection at mid gestation with a higher number of infected and damaged fetuses (**Figs 5 and 6**) is the new finding suggesting a broader period of susceptibility. More efficient transplacental spread at earlier pregnancy stages may be the common feature of different flaviviruses in mammalian species because the discovered JEV phenotype is similar to the known Zika phenotype where the virus more readily crosses the placental barrier at early pregnancy in humans and experimental animals [53,60]. The limitation in the present study is that all pig subgroups representing different pregnancy stages had only two animals, and we could not increase the group size in this study. Comparative experiments with larger animal groups are needed to confirm and better understand the kinetics of transplacental JEV infection at different stages of pregnancy.

To summarize, there are emerging concerns about vector-free sexual and transplacental flavivirus transmissions, which may change flavivirus epidemiology and expand the geographical range to territories with no insect vectors. Japanese encephalitis virus is an emerging and geographically expanding flavivirus with a history of transplacental infections in humans. Here, we discovered that JEV persists in the vaginal epithelium and has the potential for sexual transmission in humans. We also contributed to a better understanding of JEV pathogenesis during transplacental infection and showed cellular targets and persistence in the endometrium and placenta. Further studies are needed to better understand the interactions of JEV with reproductive tissues, how persistent infection affects female reproductive functions, and the risks for sexual and transplacental transmissions.

## Supporting information

S1 Fig Immunohistochemistry specific for the JEV envelope protein in primary human vaginal epithelial cells. Red staining shows JEV-positive cells. **MOI**: multiplicity of infection.
(TIF)

S2 Fig Cytopathic effect in primary human vaginal epithelial cells infected with JEV. **MOI**: multiplicity of infection.
(TIF)

S3 Fig Immunohistochemistry specific for the JEV envelope protein in primary human endometrial epithelial cells. Red staining shows JEV-positive cells. **MOI**: multiplicity of infection.
(TIF)

S4 Fig Cytopathic effect in primary human endometrial epithelial cells infected with JEV. **MOI**: multiplicity of infection.
(TIF)

S5 Fig Immunohistochemistry specific for the JEV envelope protein in the human trophoblast. Red staining shows JEV-positive cells. **MOI**: multiplicity of infection.
(TIF)

S6 Fig Cytopathic effect in the human trophoblast infected with JEV. **MOI**: multiplicity of infection. Arrowheads show focal trophoblast detachment, diffuse trophoblast detachment, and disrupted trophoblast monolayer.
(TIF)

S7 Fig Antibody and IFN-α responses in blood plasma of adult pigs. Neutralizing (**A**) and IgG virus-binding (**B**) antibody titers in non-pregnant and pregnant pigs. (**C**) Individual IFN-α concentrations in the blood plasma of non-pregnant and all pregnant pigs. Dots represent individual pigs. The dotted line is the detection limit. Columns represent mean values with standard deviations. **Ab**: Antibodies. **IPMA**: Immunoperoxidase monolayer assay.
(TIF)

S8 Fig Japanese encephalitis virus persists in different porcine tissues sampled at 28 days after inoculation. (**A**) JEV RNA loads determined in tonsils by virus-specific RT-qPCR. (**B**) JEV negative-strand RNA PCR values in tonsils. (**C**) Infectious JEV titers in tonsils determined by the endpoint dilution assay in C6/36 cells. (**D**) Mock-inoculated control C6/36 cells with no staining; (**E-F**) JEV-positive staining (red) in C6/36 cells inoculated with tonsils collected from an early pregnancy pig. (**G**) JEV RNA loads determined by virus-specific RT-qPCR in lymphoid, nervous (in the brain, replicate tissues collected from two anatomical locations—frontal and occipital lobes—were tested in each pig), respiratory, and reproductive tissues. JEV negative-strand RNA was identified in mesenteric lymph nodes. In all graphs, dots represent individual pigs. The dotted line (except in JEV negative-strand RNA PCR) is the detection limit. Columns represent mean values with standard deviations. **NA**: not available.
(TIF)

S9 Fig Fetal pathology. **(A)** A fetus with no gross pathology from early pregnancy pig A. **(B)** A fetus with edema from early pregnancy pig A. **(C)** A fetus with mild edema from mid pregnancy pig C. **(D)** A fetus with severe edema from mid-pregnancy pig C.
(TIF)

S1 Table RNA-seq. (XLS)

S2 Table Fetal pathology. (XLSX)
