# Supplementary figures and images for: Japanese encephalitis virus persists in the human reproductive epithelium and porcine reproductive tissues
Source: PLoS Negl Trop Dis. 2022 Jul 29;16(7):e0010656. doi: 10.1371/journal.pntd.0010656 (PMC9337681; doi:10.1371/journal.pntd.0010656)

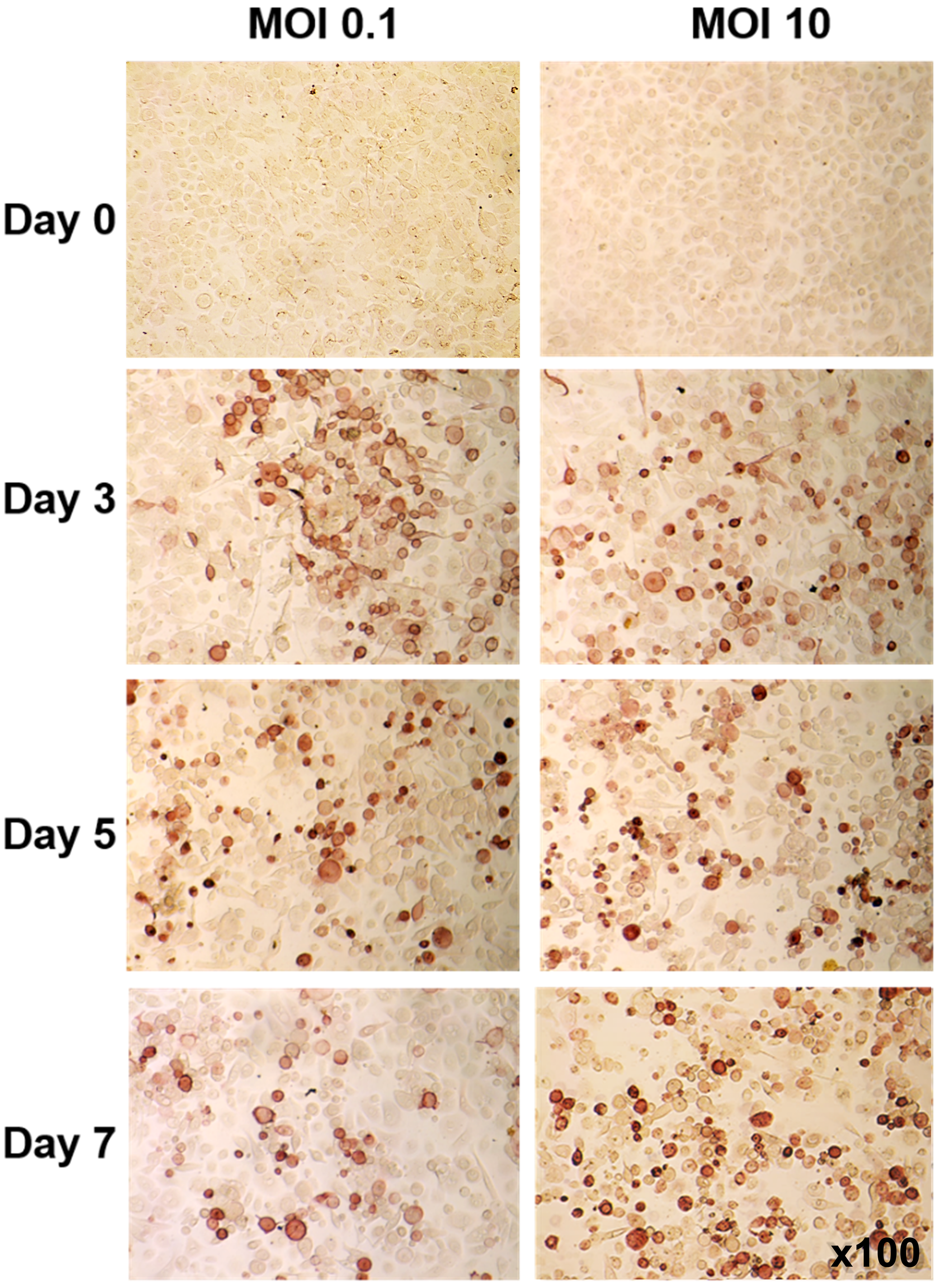

Supplement: S1 Fig — Red staining shows JEV-positive cells. MOI: multiplicity of infection. (TIF) [file pntd.0010656.s001.tif]

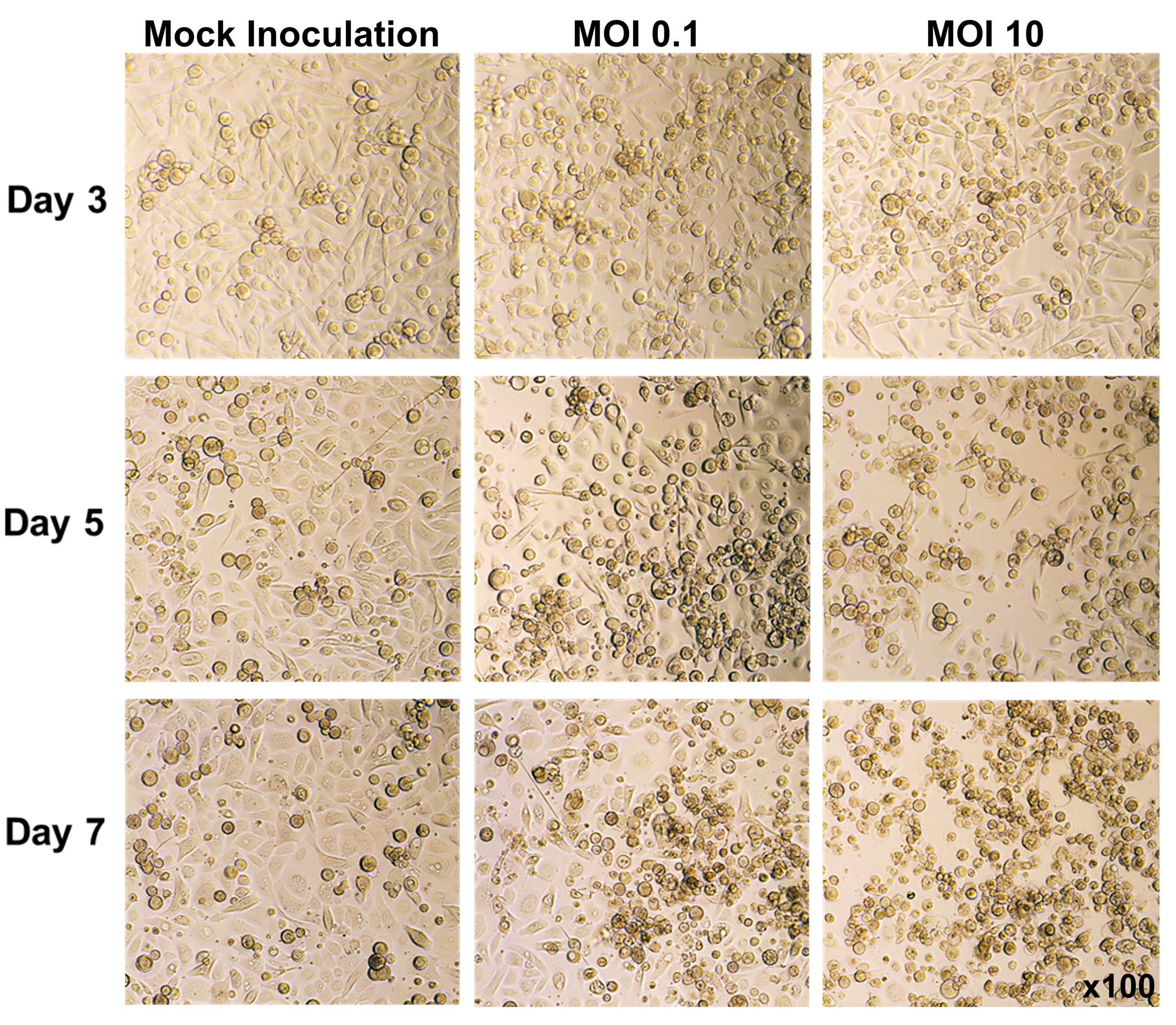

Supplement: S2 Fig — MOI: multiplicity of infection. (TIF) [file pntd.0010656.s002.tif]

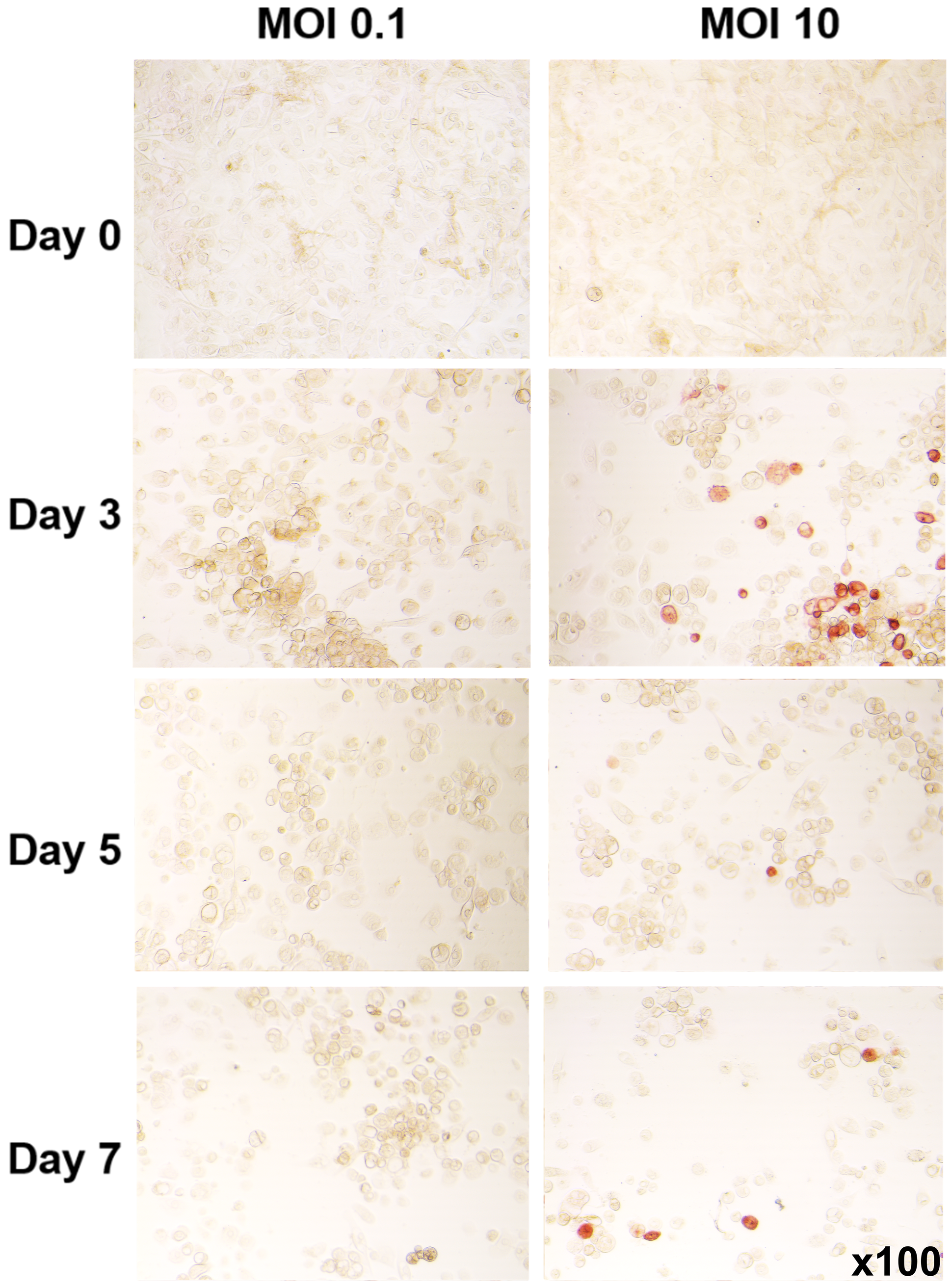

Supplement: S3 Fig — Red staining shows JEV-positive cells. MOI: multiplicity of infection. (TIF) [file pntd.0010656.s003.tif]

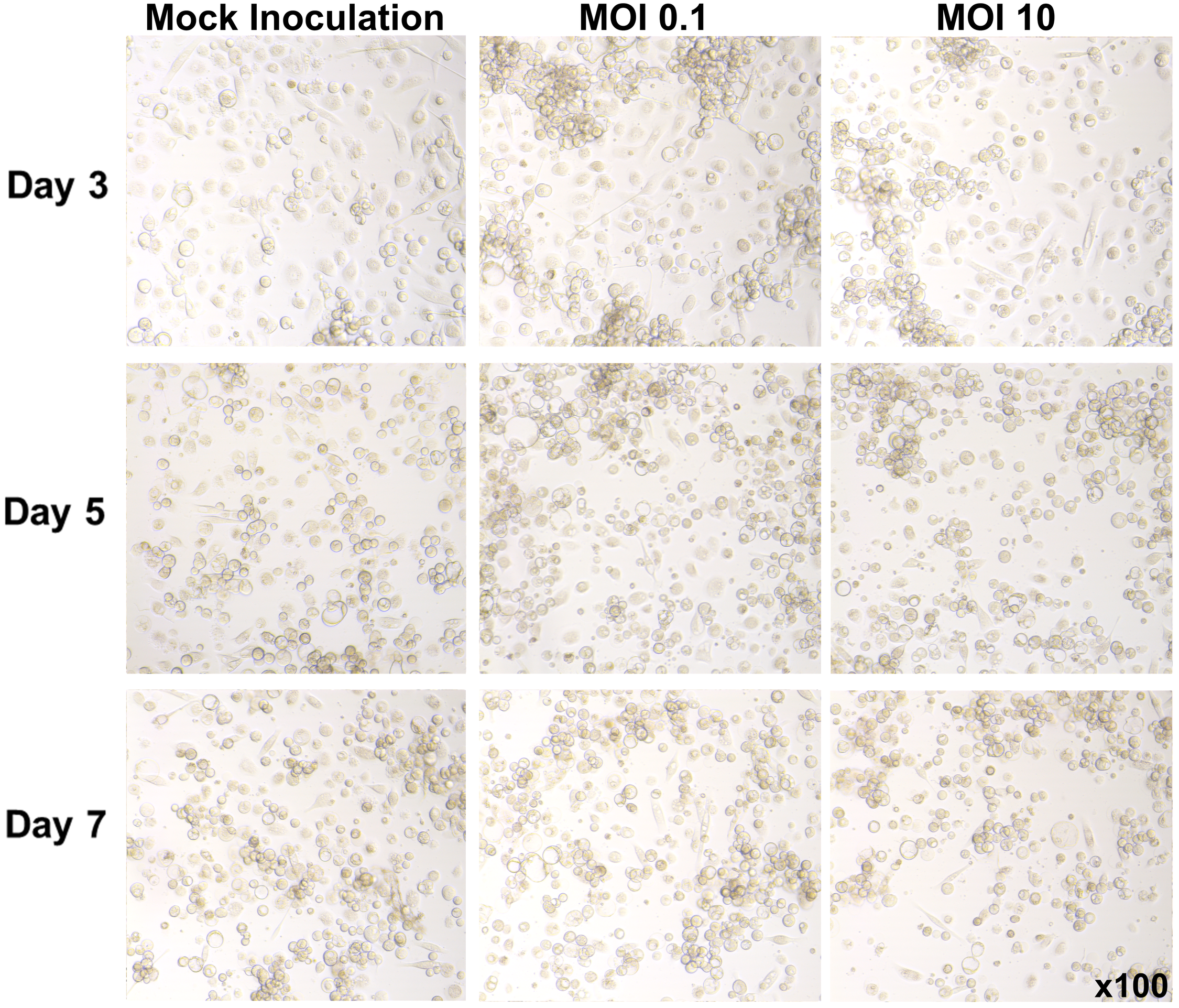

Supplement: S4 Fig — MOI: multiplicity of infection. (TIF) [file pntd.0010656.s004.tif]

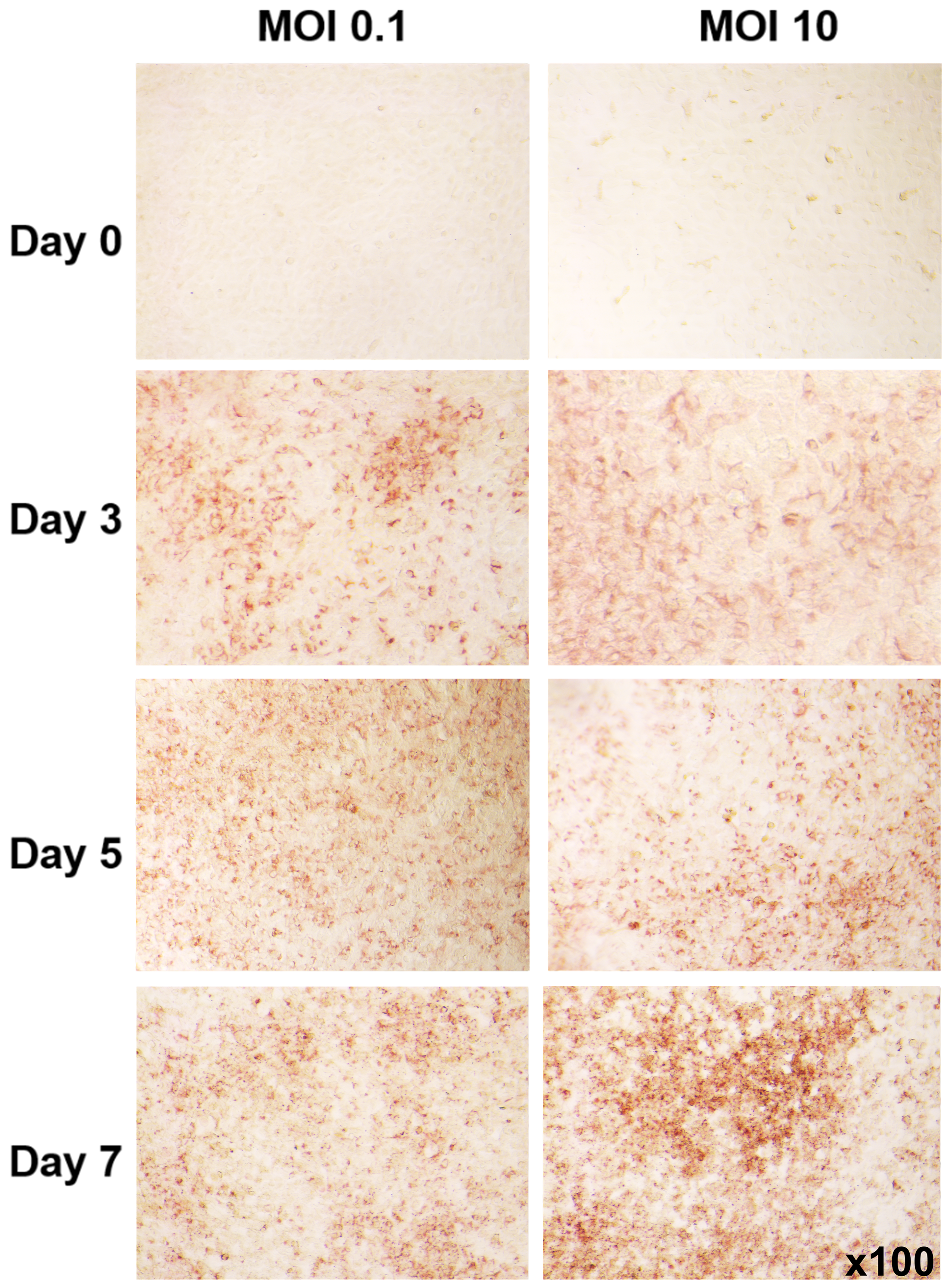

Supplement: S5 Fig — Red staining shows JEV-positive cells. MOI: multiplicity of infection. (TIF) [file pntd.0010656.s005.tif]

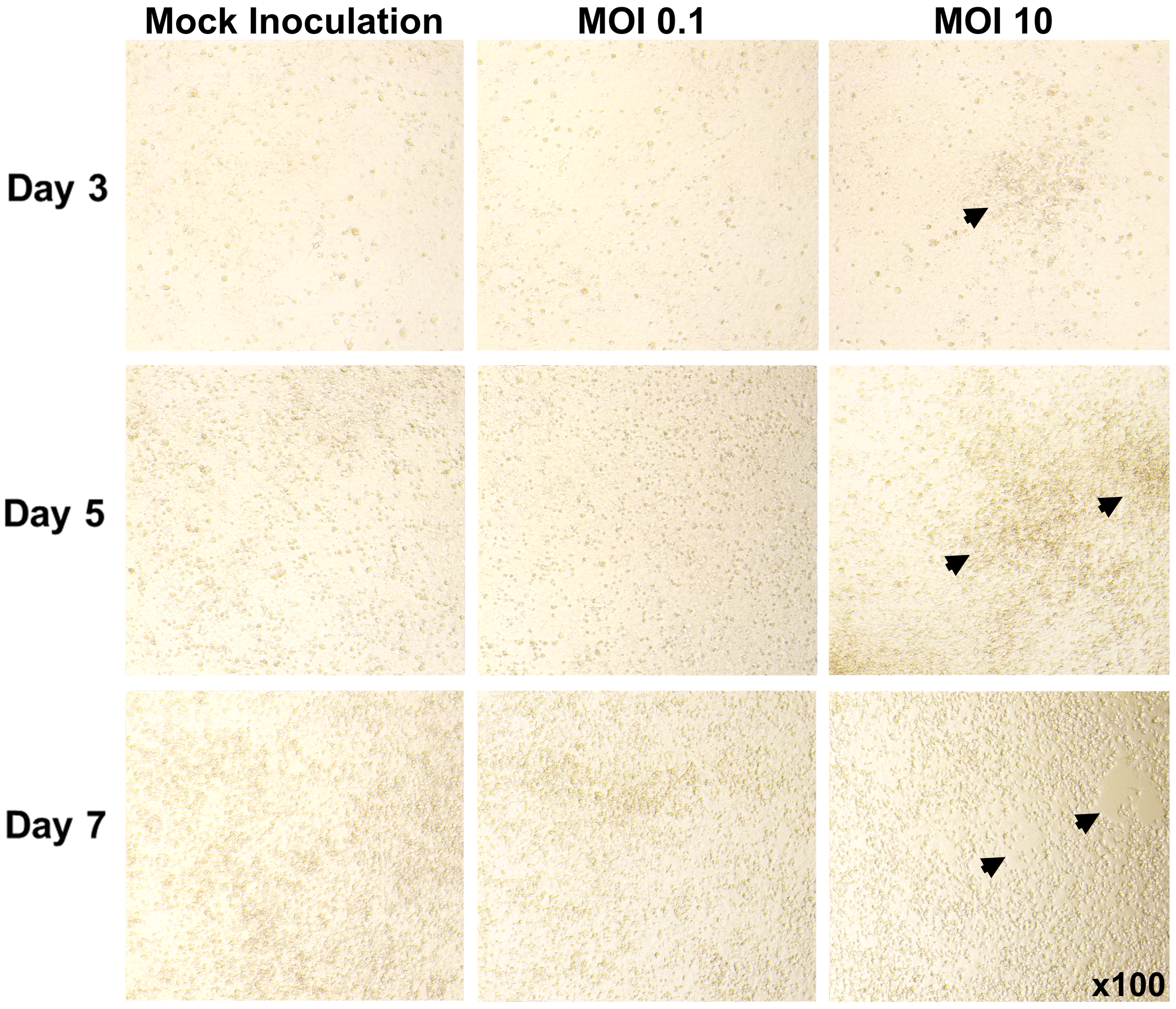

Supplement: S6 Fig — MOI: multiplicity of infection. Arrowheads show focal trophoblast detachment, diffuse trophoblast detachment, and disrupted trophoblast monolayer. (TIF) [file pntd.0010656.s006.tif]

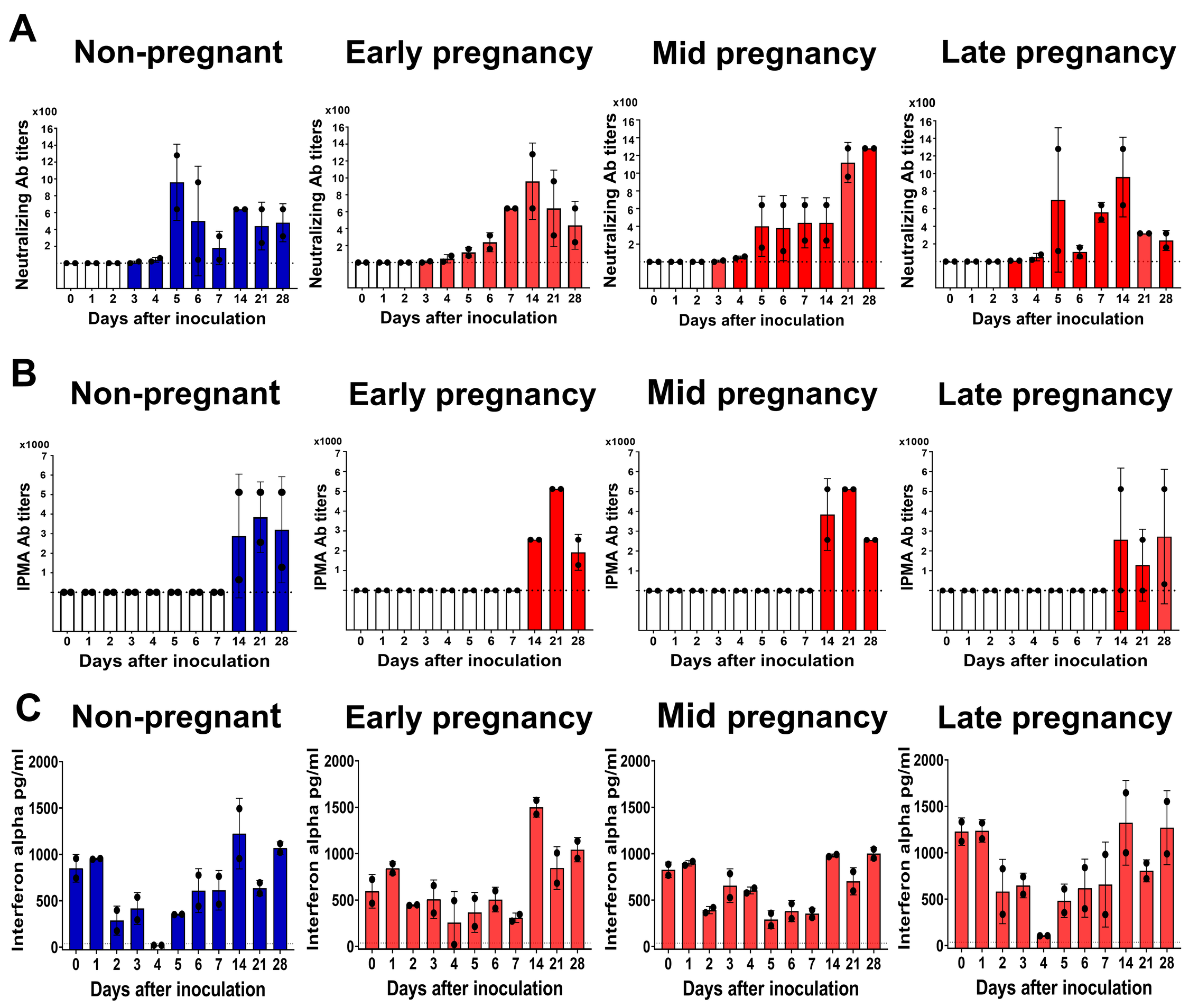

Supplement: S7 Fig — Neutralizing (A) and IgG virus-binding (B) antibody titers in non-pregnant and pregnant pigs. (C) Individual IFN-α concentrations in the blood plasma of non-pregnant and all pregnant pigs. Dots represent individual pigs. The dotted line is the detection limit. Columns represent mean values with standard deviations. Ab: Antibodies. IPMA: Immunoperoxidase monolayer assay. (TIF) [file pntd.0010656.s007.tif]

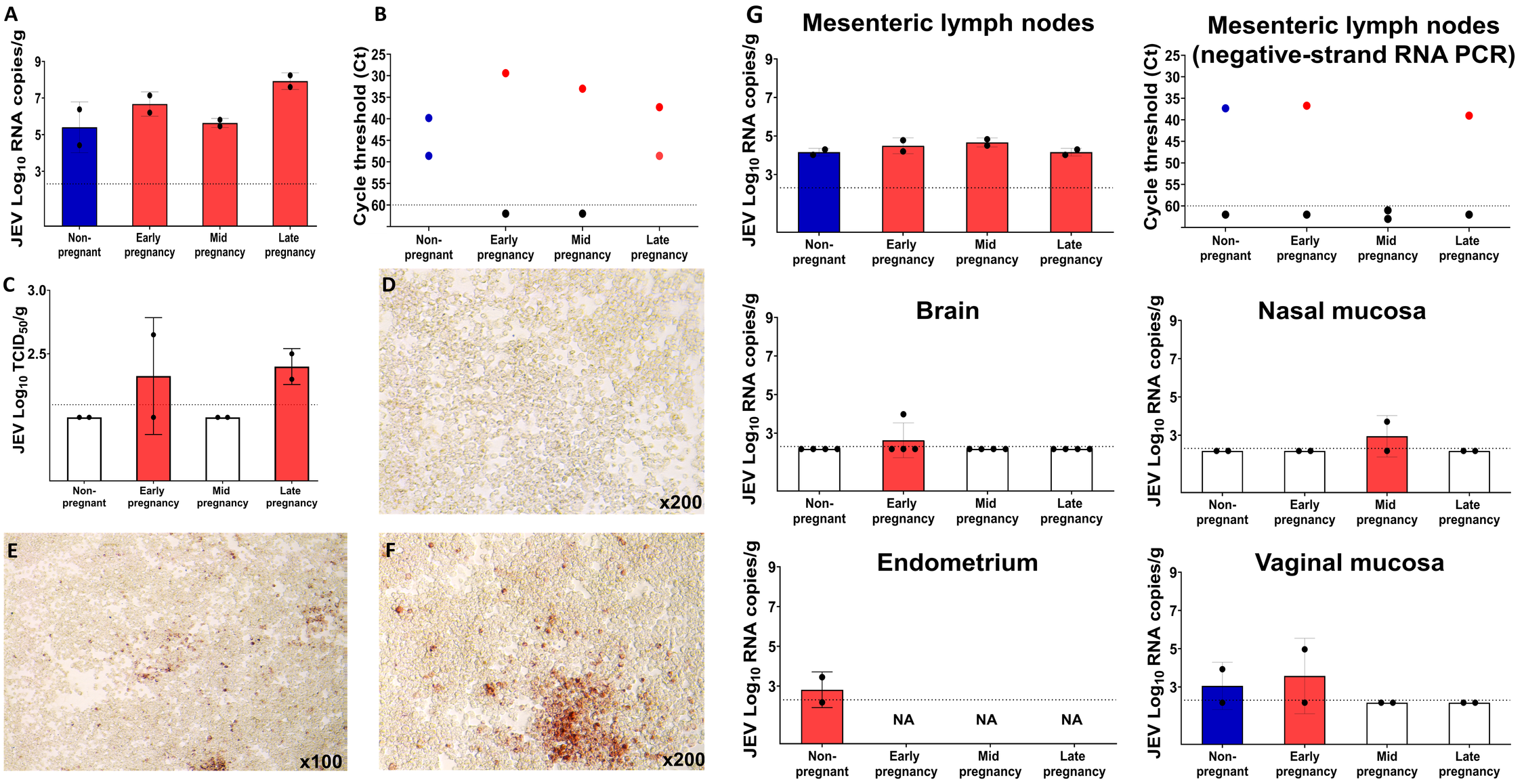

Supplement: S8 Fig — (A) JEV RNA loads determined in tonsils by virus-specific RT-qPCR. (B) JEV negative-strand RNA PCR values in tonsils. (C) Infectious JEV titers in tonsils determined by the endpoint dilution assay in C6/36 cells. (D) Mock-inoculated control C6/36 cells with no staining; (E-F) JEV-positive staining (red) in C6/36 cells inoculated with tonsils collected from an early pregnancy pig. (G) JEV RNA loads determined by virus-specific RT-qPCR in lymphoid, nervous (in the brain, replicate tissues collected from two anatomical locations—frontal and occipital lobes—were tested in each pig), respiratory, and reproductive tissues. JEV negative-strand RNA was identified in mesenteric lymph nodes. In all graphs, dots represent individual pigs. The dotted line (except in JEV negative-strand RNA PCR) is the detection limit. Columns represent mean values with standard deviations. NA: not available. (TIF) [file pntd.0010656.s008.tif]

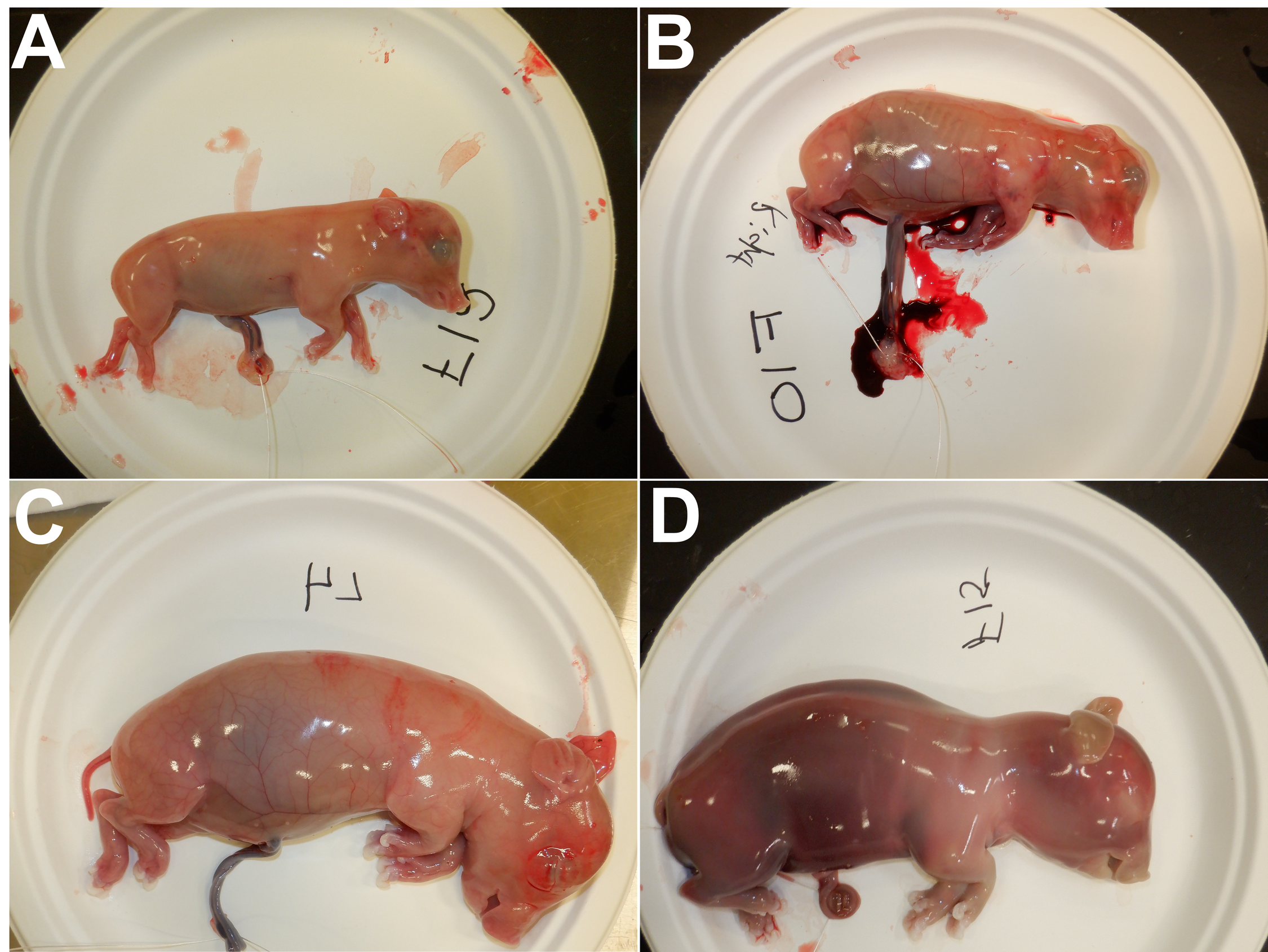

Supplement: S9 Fig — (A) A fetus with no gross pathology from early pregnancy pig A. (B) A fetus with edema from early pregnancy pig A. (C) A fetus with mild edema from mid pregnancy pig C. (D) A fetus with severe edema from mid-pregnancy pig C. (TIF) [file pntd.0010656.s009.tif]
